# Molecular species selectivity of lipid transport creates a mitochondrial sink for di‐unsaturated phospholipids

**DOI:** 10.15252/embj.2020106837

**Published:** 2021-12-07

**Authors:** Mike F Renne, Xue Bao, Margriet WJ Hokken, Adolf S Bierhuizen, Martin Hermansson, Richard R Sprenger, Tom A Ewing, Xiao Ma, Ruud C Cox, Jos F Brouwers, Cedric H De Smet, Christer S Ejsing, Anton IPM de Kroon

**Affiliations:** ^1^ Membrane Biochemistry & Biophysics Department of Chemistry Utrecht University Utrecht The Netherlands; ^2^ Department of Biochemistry and Molecular Biology VILLUM Center for Bioanalytical Sciences University of Southern Denmark Odense Denmark; ^3^ Biochemistry and Cell Biology Department of Veterinary Medicine Utrecht University Utrecht The Netherlands; ^4^ Cell Biology and Biophysics Unit European Molecular Biology Laboratory Heidelberg Germany; ^5^ Present address: Sir William Dunn School of Pathology University of Oxford Oxford UK; ^6^ Present address: Department of Medical Microbiology Radboud University Medical Center Radboud Institute for Molecular Life Sciences Nijmegen The Netherlands; ^7^ Present address: Wageningen Food & Biobased Research Wageningen University & Research Wageningen The Netherlands; ^8^ Present address: Center for Molecular Medicine University Medical Center Utrecht Utrecht The Netherlands

**Keywords:** lipid transport, membrane contact sites, membrane lipid homeostasis, membrane lipid unsaturation, mitochondria, Membranes & Trafficking, Metabolism

## Abstract

Mitochondria depend on the import of phospholipid precursors for the biosynthesis of phosphatidylethanolamine (PE) and cardiolipin, yet the mechanism of their transport remains elusive. A dynamic lipidomics approach revealed that mitochondria preferentially import di‐unsaturated phosphatidylserine (PS) for subsequent conversion to PE by the mitochondrial PS decarboxylase Psd1p. Several protein complexes tethering mitochondria to the endomembrane system have been implicated in lipid transport in yeast, including the endoplasmic reticulum (ER)‐mitochondrial encounter structure (ERMES), ER‐membrane complex (EMC), and the vacuole and mitochondria patch (vCLAMP). By limiting the availability of unsaturated phospholipids, we created conditions to investigate the mechanism of lipid transfer and the contributions of the tethering complexes *in vivo*. Under these conditions, inactivation of ERMES components or of the vCLAMP component Vps39p exacerbated accumulation of saturated lipid acyl chains, indicating that ERMES and Vps39p contribute to the mitochondrial sink for unsaturated acyl chains by mediating transfer of di‐unsaturated phospholipids. These results support the concept that intermembrane lipid flow is rate‐limited by molecular species‐dependent lipid efflux from the donor membrane and driven by the lipid species’ concentration gradient between donor and acceptor membrane.

## Graphical abstract

Mitochondrial lipid import at organelle contact sites is phospholipid molecular species‐selective, enriching mitochondria in unsaturated lipids.

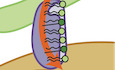

## Introduction

**Figure 1:**
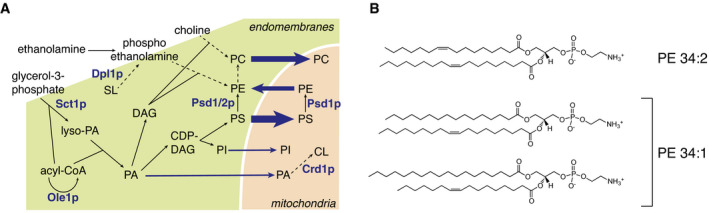
Phospholipid metabolic pathways and relevant phospholipid structures Overview of yeast phospholipid biosynthesis in the endomembrane system and mitochondria, highlighting the enzymes most relevant to this study. Dashed arrows indicate multistep reactions; blue arrows indicate lipid flow into and from mitochondria, with thickness reflecting the estimated flux. CL, cardiolipin; DAG, diacylglycerol; PA, phosphatidic acid; PC, phosphatidylcholine; PE, phosphatidylethanolamine; PI, phosphatidylinositol; PS, phosphatidylserine; SL, sphingolipids.Structures of the abundant phosphatidylethanolamine molecular species 34:2 (*sn‐1*‐palmitoleoyl‐*sn*‐2‐oleoyl‐phosphatidylethanolamine), and 34:1 (top: *sn‐1*‐palmitoyl‐*sn*‐2‐oleoyl‐phosphatidylethanolamine; bottom: *sn*‐1‐stearoyl‐*sn*‐2‐palmitoleoyl‐phosphatidylethanolamine). Molecular species are indicated by the sum of carbon atoms in the acyl chains : sum of double bonds in the acyl chains. The reader should note that MS^1^ analysis as used in this study does not reveal the identity of the phospholipid acyl chains. Therefore, the most probable acyl chain compositions based on cellular acyl chain composition (see forthcoming Fig 4) have been depicted.

Mitochondria are essential cell organelles required for a plethora of functions, including energy production and lipid metabolism. Whereas most enzymes catalyzing the synthesis of bulk membrane lipids are localized to the endoplasmic reticulum (ER), evolution left PS decarboxylase (PSD), converting phosphatidylserine (PS) to phosphatidylethanolamine (PE), in mitochondria (Fig 1A; van Meer & de Kroon, 2011). Accordingly, PE is enriched in mitochondrial membranes compared to other organelles (Tuller *et al*, 1999). Mitochondria also harbor the enzymes involved in the biosynthesis of the mitochondrial signature lipid cardiolipin (CL) (Schlame & Greenberg, 2017). For the synthesis of PE and CL, mitochondria depend on import of the respective lipid precursors PS and phosphatidic acid (PA) from the ER (Dimmer & Rapaport, 2017; Acoba *et al*, 2020).

The importance of PE and CL in mitochondrial function is well established (recently reviewed in Mårtensson *et al*, 2017; Basu Ball *et al*, 2018; Acoba *et al*, 2020). PE has been detected in the crystal structures of respiratory complexes (Lange *et al*, 2001; Shinzawa‐Itoh *et al*, 2007; Guo *et al*, 2017) and was shown to be required for complex III and IV activity (Baker *et al*, 2016; Calzada *et al*, 2019). Local biosynthesis of PE in mitochondria is required for optimal respiration in yeast (Birner *et al*, 2001; Friedman *et al*, 2018; Calzada *et al*, 2019), and for potentiating respiratory capacity of skeletal muscles (Heden *et al*, 2019), underscoring the importance of the mitochondrial localization of PS decarboxylase. Mitochondrial PE has also been implicated in the regulation of mitochondrial fusion via Mgm1 (Osman *et al*, 2009; Chan & McQuibban, 2012), and in the biogenesis of outer mitochondrial membrane beta‐barrel proteins (Becker *et al*, 2013). Furthermore, defective PS transfer to mitochondria inhibiting mitochondrial PE synthesis leads to liver disease (Hernández‐Alvarez *et al*, 2019).

Both PE and CL are membrane lipids with non‐bilayer propensity that confers negative intrinsic curvature to membranes, the extent increasing with the content of unsaturated acyl chains and their length (Renne & de Kroon, 2018). The enrichment of PE and CL in unsaturated acyl chains (Schneiter *et al*, 1999; Ejsing *et al*, 2009) further supports this notion. Loss of mitochondrial synthesis of PE and CL is synthetically lethal in yeast (Gohil *et al*, 2005), indicating overlapping functions and inferring that a minimum level of non‐bilayer preferring lipids is essential for mitochondrial function (Calzada *et al*, 2019). Mitochondria harbor the transacylase Tafazzin (Taz1p in yeast) to maintain a high degree of unsaturation in CL by acyl chain remodeling (Schlame & Greenberg, 2017). Much less is known on how the molecular species composition of mitochondrial PE is established.

Mitochondrial PSD enzymes are evolutionarily conserved (Di Bartolomeo *et al*, 2017). In yeast, the mitochondrial PSD Psd1p catalyzes up to 90% of PS‐to‐PE conversion (Storey *et al*, 2001; Calzada *et al*, 2019), with the remaining activity provided by Psd2p that is localized in endosomes (Gulshan *et al*, 2010). Part of the PE produced in mitochondria relocates to the ER to be methylated to phosphatidylcholine (PC). As a consequence, yeast cells rely heavily on lipid transport between ER and mitochondria for supply of the major membrane lipids PE and PC, particularly in the absence of ethanolamine and choline, substrates of the CDP‐ethanolamine and CDP‐choline routes, respectively, that produce PE and PC in the ER (Fig 1A, Henry *et al*, 2012). A twist to the longstanding concept of PSD activity residing exclusively outside the ER, came with the recent finding of a dual localization of Psd1p to mitochondria and ER, the distribution depending on metabolic demand (Friedman *et al*, 2018). However, the presence of a functional subpopulation of Psd1 in the ER was not confirmed in an independent re‐evaluation (Sam *et al*, 2021), leaving it under debate.

To facilitate lipid transport, mitochondria maintain organelle contact sites (OCS) with the ER (Gaigg *et al*, 1995) and the vacuole (Dimmer & Rapaport, 2017). The ER subfraction associated with mitochondria is called mitochondria‐associated membrane (MAM) and is enriched in lipid biosynthetic activity (Gaigg *et al*, 1995; Kannan *et al*, 2017). The protein complexes that tether mitochondria to the ER, *i.e*., the ER‐mitochondrial encounter structure (ERMES; Mmm1p, Mdm10, 12, 34p regulated by Gem1p, Kornmann *et al*, 2009, 2011) and the ER membrane protein complex (EMC; Emc1‐6p, Lahiri *et al*, 2014), as well as those that tether the ER to the vacuole, *i.e*., Vps13p‐Mcp1p, and the vacuole and mitochondria patch (vCLAMP; Vps39p, Ypt7p, and Tom40p, Elbaz‐Alon *et al*, 2014; Hönscher *et al*, 2014; González Montoro *et al*, 2018; John Peter *et al*, 2017; Park *et al*, 2016) have been implicated in lipid transport, of PS in particular. Recently, both a heterodimer of the ERMES subunits Mmm1p and Mdm12p (Jeong *et al*, 2017; Kawano *et al*, 2018), and the vacuole contact site protein Vps13p (Kumar *et al*, 2018) were shown to mediate lipid transfer *in vitro*. However, the contribution of each tethering complex to lipid transport, and the molecular details of the process remain elusive.

Here, we address the role of mitochondrial lipid import in establishing the cellular lipid composition. A dynamic lipidomics approach uncovered that PS‐to‐PE conversion mainly produces di‐unsaturated PE molecular species (*i.e*., containing two unsaturated acyl chains, Fig 1B), a preference that is dependent on the mitochondrial localization of Psd1p. These results indicate that lipid transport is molecular species selective, account for the enrichment of di‐unsaturated species in (mitochondrial) PE, and explain why mitochondria are a sink for unsaturated acyl chains. The mechanism of mitochondrial lipid import including the role of OCS was interrogated by manipulating the cellular level of lipid unsaturation through overexpression of *SCT1*. *SCT1* encodes a glycerol‐3‐phosphate acyltransferase (Fig 1A) that prefers C16:0‐CoA as substrate (Zheng & Zou, 2001). Overexpression of *SCT1* sequesters C16:0 acyl chains in glycerolipids, protecting them from desaturation by the single Δ9‐desaturase Ole1p, thus increasing the cellular content of saturated (SFA) at the expense of unsaturated acyl chains (UFA) (De Smet *et al*, 2012). Under these conditions, inactivation of ERMES or Vps39p impairs mitochondrial lipid import, reducing the mitochondrial sink for UFA. The findings indicate that ERMES and Vps39p facilitate species‐selective mitochondrial lipid import *in vivo*. Moreover, the results argue that tethering complexes serve as passive conduits for concentration gradient‐driven lipid transport.

## Results

### PS decarboxylation is molecular species selective

To investigate PS‐to‐PE conversion by Psd1p at the level of individual lipid molecules, the incorporation of stable isotope‐labeled ^13^C_3_^15^N‐serine into PS and the subsequent decarboxylation to ^13^C_2_^15^N‐PE was monitored after a 20‐min pulse by high‐resolution mass spectrometry in an established set of PSD mutants (Friedman *et al*, 2018). In the mutants, Psd1p was either inactivated or left as the only source of cellular PE by deletion of *PSD2* and *DPL1*, the latter encoding a dihydrosphingosine lyase that generates phosphoethanolamine (Gottlieb *et al*, 1999), an intermediate in the CDP‐ethanolamine route (Fig 1A). Strains were cultured in synthetic defined galactose medium (SGal) devoid of choline and ethanolamine to confer maximal dependence on PS decarboxylation for *de novo* synthesis of phospholipids. The non‐preferred carbon source galactose chosen to enable expression from the *GAL* promoter in the forthcoming, rendered cells partly dependent on mitochondria for energy supply (Barford & Hall, 1979; Morgenstern *et al*, 2017).

**Figure 2:**
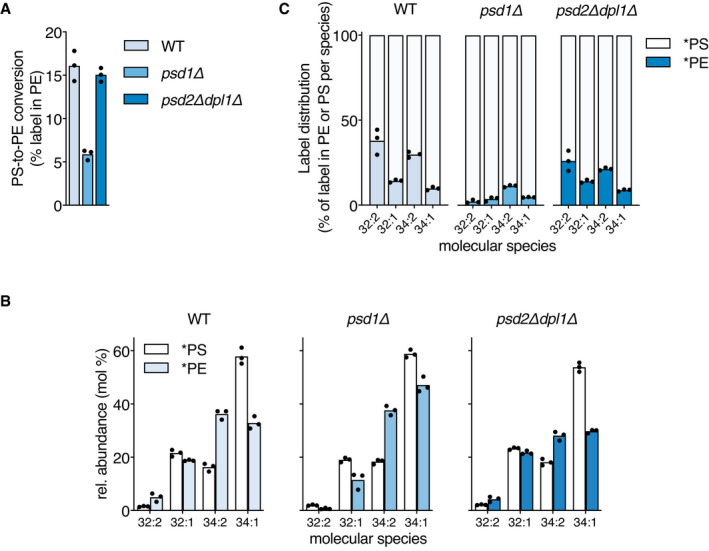
Mitochondrial PS decarboxylation by Psd1p prefers di‐unsaturated PS molecular species A, BPS decarboxylase activity in wild‐type (WT, W303) and the indicated mutant strains expressed as (A) percentage of the lipid‐incorporated ^13^C^15^N‐label recovered in PE, and (B) the molecular species composition of ^13^C_3_^15^N‐labeled PS (*PS) and ^13^C_2_^15^N‐labeled PE (*PE), after 20‐min incubation with ^13^C_3_^15^N‐serine.CThe corresponding molecular signatures of PSD activity represented by the label distribution per molecular species between *PS and *PE as percentages of the total amount of label incorporated in the molecular species indicated. Data information: Exponentially growing cells were pulsed for 20 min with ^13^C_3_^15^N‐serine at 30°C prior to lipid extraction and lipid analysis by shotgun lipidomics. Molecular species representing at least 1% of both labeled PS and labeled PE are shown in panel B (as mol% of total). Compared to *PS and *PE, the amount of ^13^C_2_^15^N ‐PC synthesized was negligible (*cf*. Elbaz‐Alon *et al*, 2014) and therefore not included in the analysis. Data are presented as the mean of 3 biological replicates, with the individual values indicated. Underlying data for this figure can be found in Dataset EV1.

The growth phenotype on SGal of the *psd1Δ* and *psd2Δdpl1Δ* mutants compared to WT was similar to that on synthetic defined glucose medium (SD) (Appendix Fig S1A) (Friedman *et al*, 2018), with *psd1Δ* showing delayed growth. Analysis of stable isotope‐labeled PS (*PS) and PE (*PE) abundance after the 20‐min pulse showed that PE synthesis is decreased by ± 66% in *psd1Δ* compared to WT, whereas in *psd2Δdpl1Δ* in which Psd1p activity is the only source of PE, PS‐to‐PE conversion was similar to WT (Fig 2A). Western blot analysis (Appendix Fig S1B) showed similar Psd1p expression levels in WT and *psd2Δdpl1Δ*, arguing that Psd1p is the main source of PE in WT under the conditions used. In WT cells, the proportions of the di‐unsaturated molecular species *PE 32:2 and *PE 34:2 (for an explanation of lipid nomenclature, see Fig 1B and legend) are enriched in newly synthesized PE compared to the proportions of the corresponding species in newly synthesized PS (Fig 2B and C). The enrichment of di‐unsaturated *PE is at the expense of the mono‐unsaturated 34:1 species that shows a lower proportion in *PE than in *PS (Fig 2B and C). In *psd1Δ*, the preferential decarboxylation of *PS 34:2 was retained, whereas the enrichment of *PE 32:2 was lost and compensated for by a rise in the proportion of *PE 34:1. The enrichment of the di‐unsaturated *PE species in *psd2Δdpl1Δ* was similar to WT (Fig 2B), in agreement with Psd1p being the main PS decarboxylase (Trotter & Voelker, 1995; Birner *et al*, 2001; Storey *et al*, 2001).

In line with these observations, steady‐state PE and PC in WT and *psd2Δdpl1Δ* were enriched in di‐unsaturated species compared to the steady state PS, mainly at the expense of 34:1. This enrichment was largely lost in *psd1Δ* (Appendix Fig S2).

Taken together, the data show that preferential decarboxylation of di‐unsaturated PS species by Psd1p plays a major role in establishing the PE and PC molecular species profiles under the culture conditions applied.

### Molecular species selectivity of mitochondrial PS decarboxylation is due to species‐selective transport rather than Psd1p substrate specificity

The observed molecular species‐selective conversion of PS by Psd1p could originate from substrate specificity, or from molecular species‐dependent lipid transport as proposed previously (Heikinheimo & Somerharju, 1998, 2002). To distinguish between these possibilities, we investigated the molecular species selectivity of PS‐to‐PE conversion in *psd1Δpsd2Δdpl1Δ* cells expressing chimeric Psd1p constructs that localize exclusively to the ER (Sec66‐Psd1p chimera; +Psd1(ER)) or to mitochondria (Mic60‐Psd1p chimera; +Psd1(Mt)) in comparison with WT Psd1p (+Psd1(WT)). The constructs are expressed from the native *PSD1* promoter and stably integrated in the genome (Friedman *et al*, 2018). Growth of the strains expressing Psd1 constructs on SGal was similar to that on SD, with delayed growth of the +Psd1(Mt) (Appendix Fig S1A) as previously observed (Friedman *et al*, 2018). Western blot analysis verified the expression of the Psd1 constructs in *psd1Δpsd2Δdpl1Δ* on SGal (Appendix Fig S1B).

**Figure 3:**
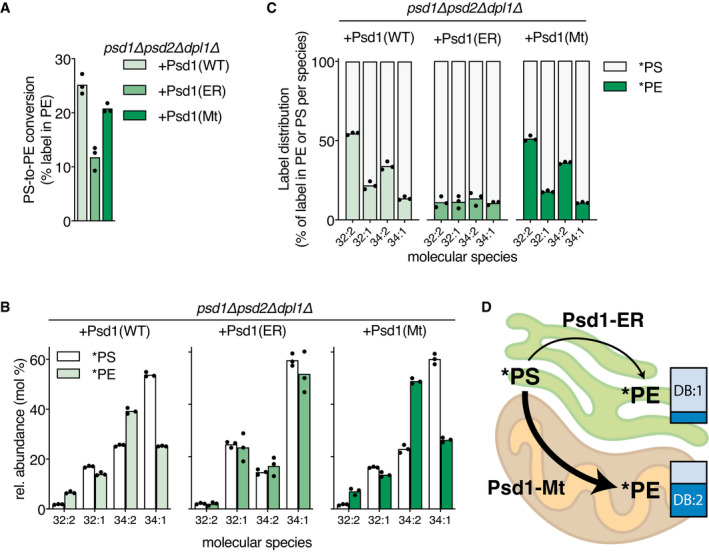
Molecular species selectivity of Psd1 depends on its localization in mitochondria A–CPS decarboxylase activity in a *psd1Δpsd2Δdpl1Δ* mutant expressing Psd1(WT), Psd1(ER) or Psd1(Mt) expressed as (A) percentage of the lipid‐incorporated ^13^C^15^N‐label recovered in PE, and (B) the molecular species composition of *PS and *PE after 20 min incubation with ^13^C_3_^15^N‐serine; (C) the corresponding molecular signatures of PSD activity.DCartoon depicting the enrichment of di‐unsaturated PE molecular species produced by Psd1(Mt) versus Psd1(ER), with the thickness of the arrows reflecting relative enzyme activities. Based on the data presented in panels A and B; DB:1 and DB:2, fractions of *PE molecular species with 1 and 2 double bonds, respectively. Data information: For experimental details see the legend to Fig 2. Data are presented as the mean of 3 biological replicates, with the individual values indicated. Underlying data for this figure can be found in Dataset EV1.

Stable isotope labeling showed that PS‐to‐PE conversion proceeded at a higher rate in *psd1Δpsd2Δdpl1Δ* cells expressing Psd1(WT) (Fig 3A) compared with WT and *psd2Δdpl1Δ* strains expressing endogenous Psd1p (Fig 2A), consistent with the respective Psd1p expression levels (Appendix Fig S1B). PS‐to‐PE conversion was similar in *psd1Δpsd2Δdpl1Δ* cells expressing Psd1(WT) or Psd1(Mt), whereas it was decreased in cells expressing Psd1(ER) (Fig 3A). Expression of the Psd1(ER) construct was lower than of Psd1(WT) for reasons presently not understood, possibly explaining lower PSD activity. Previously, Psd1(ER) was reported to exhibit reduced activity under conditions where expression was similar to Psd1(WT) or Psd1(Mt) (Friedman *et al*, 2018). Expression of the Psd1(Mt) construct was slightly higher than that of theIIIII wild‐type Psd1 construct (Appendix Fig S1B; in agreement with Friedman *et al*, 2018), yet PS‐to‐PE conversion in +Psd1(Mt) was not increased compared to +Psd1(WT) (Fig 3A, Friedman *et al*, 2018), indicating that the expression level of mitochondrial Psd1p is not rate‐limiting in +Psd1(WT) cells.

The *psd1Δpsd2Δdpl1Δ* strains expressing Psd1(WT) and Psd1(Mt) shared similar species profiles of newly synthesized PS and PE with a preference for the conversion of *PS 32:2 and *PS 34:2 to *PE (Fig 3B), as was observed in WT and *psd2Δdpl1Δ* (Fig 2B). Strikingly, in the *psd1Δpsd2Δdpl1Δ* strain expressing Psd1(ER), the *PS molecular species are converted to *PE with similar efficiencies (Fig 3B), demonstrating that the molecular species selectivity of PS decarboxylation depends on the mitochondrial localization of Psd1p. Moreover, Psd1p did not show any molecular species preference in decarboxylating endogenous and exogenously added PS molecular species in detergent‐solubilized mitochondria (Appendix Fig S3), supporting Psd1p’s lack of intrinsic substrate preference.

The distribution of the amount of label incorporated per molecular species between PS and PE provides the molecular signature of PSD (Figs 2C and 3C). The PSD signature of WT is similar to that of *psd2Δdpl1Δ*, and both differ from that in *psd1Δ*, in agreement with Psd1p contributing the larger share of newly produced PE in WT (Fig 2A) (Trotter & Voelker, 1995; Birner *et al*, 2001; Storey *et al*, 2001). The molecular signatures show strong resemblance between the strains in which Psd1 has a mitochondrial localization (Figs 2C and 3C). The similarity of the PSD signatures of *psd1Δpsd2Δdpl1Δ* cells expressing Psd1(WT) and Psd1(Mt) argues that the contribution of the ER‐localized Psd1p to PSD activity in the former, although essential for optimal growth (Appendix Fig S1A and Friedman *et al*, 2018), is quantitatively secondary. The differences in PS conversion between *psd1Δpsd2Δdpl1Δ* cells expressing Psd1(WT) or Psd1(Mt) on the one hand versus *psd1Δpsd2Δdpl1Δ* expressing Psd1(ER) on the other are also apparent from the steady‐state molecular species profiles with an enrichment of mono‐unsaturated (32:1 and 34:1) PS, PE, and PC in the latter (Appendix Fig S2).

The delayed growth of *psd1Δpsd2Δdpl1Δ* +Psd1(Mt) on SGal and SD (Appendix Fig S1A, Friedman *et al*, 2018) is not due to a lack of PE, since the *psd1Δpsd2Δdpl1Δ* mutants expressing Psd1(Mt) and wild‐type Psd1 have similar PE levels (Dataset EV1). Instead, Psd1(Mt) may not be able to meet the requirement for PE in the endomembrane system, as proposed by Friedman *et al* (2018). The endomembrane system may suffer from a lower PE level or from a shortage of specific PE molecular species that are insufficiently supplied from the mitochondria. The notion that a minimum of PE synthesized in the ER is required for optimal growth is supported by the recent finding that several independent Psd1 constructs that localize exclusively to the mitochondrial inner membrane do rescue the ethanolamine auxotrophy of a *psd1Δpsd2Δ* mutant in which Dpl1p supplies the CDP‐ethanolamine route with phosphoethanolamine (Sam *et al*, 2021).

Taken together, our data show that the species composition of newly produced PE is dependent on Psd1p localization (Fig 3D). We conclude that the molecular species selectivity of PS‐to‐PE conversion in yeast mitochondria is due to preferential import of di‐unsaturated PS molecular species in agreement with previous findings in mammalian cells (Heikinheimo & Somerharju, 2002).

### Inactivation of mitochondrial lipid biosynthetic enzymes enhances the accumulation of saturated acyl chains under conditions of *SCT1* overexpression

To address the molecular mechanism of mitochondrial lipid import, we limited the cellular content of unsaturated acyl chains in wild‐type and mutants impaired in mitochondrial lipid biosynthesis and import, available in the BY4741 background. Overexpression of *SCT1* was previously shown to increase the cellular content of saturated acyl chains (SFA), C16:0 in particular (De Smet *et al*, 2012). Episomal overexpression of *SCT1* from a *GAL1* promoter accordingly increased the mono‐unsaturated molecular species in PS, PE, and PC (and the di‐saturated species in PS and PC), at the expense of di‐unsaturated species in the BY4741 wild‐type strain (Appendix Fig S4). Of note, compared to W303 (Appendix Fig S2), BY4741 (derived from FY1679) was enriched in C32 molecular species, consistent with the different acyl chain compositions of the two strain backgrounds (Daum *et al*, 1999).

**Figure 4:**
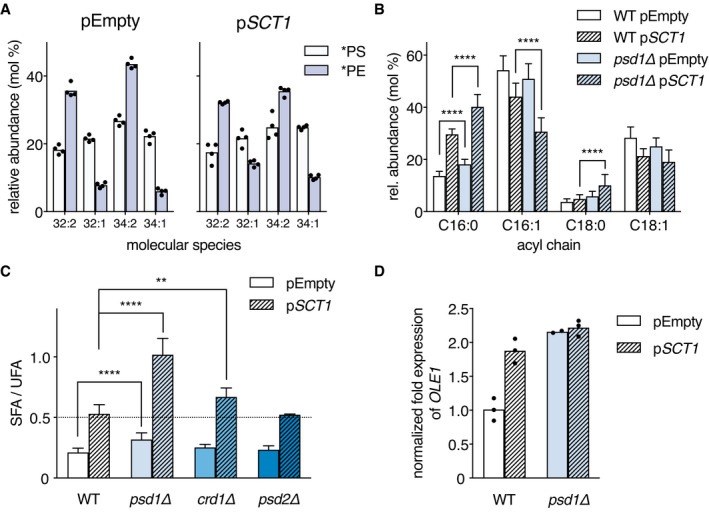
Loss of mitochondrial lipid biosynthetic enzymes enhances the accumulation of saturated acyl chains under conditions of *SCT1* overexpression The molecular species selectivity of PS‐to‐PE conversion is recapitulated in wild‐type BY4741. WT cells overexpressing *SCT1* versus control were pulsed for 20 min with ^2^H_3_‐serine prior to lipid extraction and ESI‐MS/MS analysis of ^2^H_3_‐PS (*PS) and ^2^H_3_‐PE (*PE). Data are presented as mean of 4 biological replicates with the individual values indicated. Underlying data for this figure can be found in Dataset EV2.Acyl chain composition of WT and *psd1Δ* cells overexpressing *SCT1* (p*SCT1*) versus empty vector control (pEmpty). Data is presented as mean ± SD (*n* > 10). ****P < 0.0001, multiple *t*‐test.Ratio of the proportions of saturated over unsaturated acyl chains (SFA/UFA) of WT, *psd1Δ*, *crd1Δ*, and *psd2Δ* overexpressing *SCT1* versus empty vector control. Data are presented as mean ± SD (*n* ≥ 3). **P < 0.01, ****P < 0.0001, unpaired two‐tailed *t*‐test.
*OLE1* transcript levels in WT and *psd1Δ* transformed with p*SCT1* versus pEmpty as determined by RT–qPCR. Data were normalized to endogenous *ACT1* mRNA, and to the average of WT pEmpty (*n* = 3).

Pulse labeling BY4741 with stable isotope‐labeled ^2^H_3_‐serine for 20 min revealed that, irrespective of *SCT1* overexpression, di‐unsaturated PS species were more readily converted to PE than mono‐saturated molecular species, similar as in W303 (Figs 4A and 2B), with superimposed a preference for decarboxylation of C32‐ over C34‐PS species (Fig 4A). However, in the *SCT1*‐overexpressing cells the proportion of newly synthesized di‐unsaturated PE was decreased by 15% compared to the empty vector control.

Supplementation of the SGal medium with 0.05% glucose was required to allow proper growth of mutants disturbed in mitochondrial membrane biogenesis (Appendix Fig S5A). This modification of the culture medium had only minor effect on the rise in the proportion of total SFA induced by overexpression of *SCT1* (Appendix Fig S5C). Importantly, interference with mitochondrial lipid biosynthesis by deleting *PSD1* aggravated the phenotype conferred by *SCT1* overexpression, as the proportions of C16:0 and C18:0 acyl chains were further increased at the expense of C16:1 and C18:1 (Fig 4B, Appendix Table S1), corresponding to a twofold rise of the ratio of the proportions of saturated over unsaturated acyl chains (SFA/UFA) compared to WT (Fig 4C). Accordingly, the *psd1Δ* mutant overexpressing *SCT1* had a stronger growth phenotype than WT (Appendix Fig S5B). A much smaller, yet significant rise in SFA/UFA was observed in *psd1Δ* transformed with the empty vector compared to WT (Fig 4B and C), reflecting an increase in lipid saturation independent of *SCT1* overexpression, in agreement with the rise in 34:1 level in the PE and PC profiles of the *psd1Δ* mutant in the W303 background (Appendix Fig S2). Enhancement of the accumulation of SFA induced by *SCT1* overexpression also occurred in *crd1Δ* cells that lack cardiolipin synthase, whereas it was absent in *psd2Δ* (Fig 4C), showing that the effect is not general for mutants in lipid metabolism.

The enhancement of the *SCT1* overexpression phenotype in *psd1Δ* and *crd1Δ* reflects increased incorporation of saturated acyl chains into lipids by Sct1p, outcompeting acyl‐CoA desaturation by Ole1p (De Smet *et al*, 2012). Ole1p is known to be regulated at the level of transcription in response to changes in membrane physical properties (Martin *et al*, 2007; Ballweg & Ernst, 2017). Analysis by RT–qPCR showed that the increase in SFA levels in *psd1Δ* under *SCT1* overexpression was not due to a reduction in *OLE1* expression (Fig 4D). Instead, the *OLE1* transcript level was increased under conditions of *SCT1* overexpression and/or deletion of *PSD1*, probably in response to increased acyl chain saturation (Ballweg *et al*, 2020). We speculate that feedback inhibition resulting from the diminished draw on the pool of di‐unsaturated lipids and acyl‐CoA’s caused by inactivation of Psd1p (or Crd1p) may tip the balance toward incorporation of saturated acyl chains into glycerolipids by Sct1p at the expense of desaturation by Ole1p. Conversely, the levels of newly synthesized di‐unsaturated PS and PE went up with increasing mitochondrial Psd1 activity (Appendix Fig S6), indicating that increased draw enhances the level of desaturation.

Taken together, the results define mitochondria as a sink for unsaturated acyl chains, in agreement with the above preferential conversion of di‐unsaturated PS species to PE and the established enrichment of unsaturated acyl chains in mitochondrial membranes (Tuller *et al*, 1999).

### ERMES and vCLAMP mutants overexpressing *SCT1* accumulate saturated acyl chains and exhibit a growth defect

**Figure 5:**
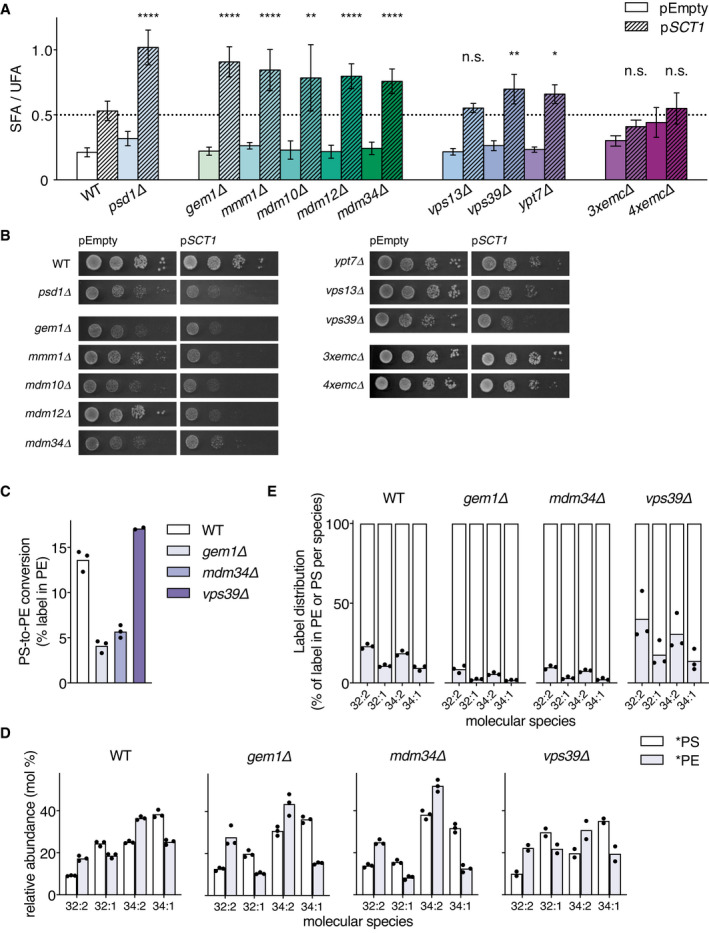
ERMES and vCLAMP mutants retain preferential decarboxylation of di‐unsaturated PS, and accumulate saturated acyl chains upon overexpressing *SCT1* ASFA/UFA ratios of WT and indicated mutant strains overexpressing *SCT1* (p*SCT1*) versus control (pEmpty). Data is presented as mean ± SD (*n* ≥ 3). *P < 0.05, **P < 0.01, ****P < 0.0001, n.s. not significant, unpaired two‐tailed *t*‐test of the indicated bar compared to WT p*SCT1*.BGrowth of WT and indicated mutant strains overexpressing *SCT1* (p*SCT1*) versus pEmpty. Serial dilutions (10^−1^–10^−4^) were spotted on SGal + 0.05% glucose and incubated for 3 days at 30°C.C, DPS decarboxylase activity in wild‐type (WT, BY4741) and the indicated mutant strains transformed with pEmpty, expressed as (C) percentage of the lipid‐incorporated ^13^C^15^N‐label recovered in PE, and (D) molecular species composition of ^13^C_3_^15^N‐labeled PS (*PS) and ^13^C_2_^15^N‐labeled PE (*PE) after 20 min incubation with ^13^C_3_^15^N‐serine. The differences in molecular species profiles of *PS and *PE between the BY4741 WT shown here and those obtained by ^2^H_3_‐serine labeling (Fig 4A) are attributed to the different culture media, lipid extraction procedures and mass spectrometry techniques used.EThe corresponding molecular signatures of PSD activity represented by the label distribution per molecular species between *PS and *PE as percentages of the total amount of label incorporated in the molecular species indicated. Data information: For experimental details, see the legend to Fig 2. Data in C, D, E are presented as the mean of 3 or 2 (*vps39Δ*) biological replicates, with the individual values indicated. Underlying data for this figure can be found in Dataset EV3.

If ERMES, EMC, and vCLAMP contribute to mitochondrial phospholipid import, mutants lacking components of these tethering complexes are predicted to exhibit a reduction of the mitochondrial UFA sink. ERMES consists of Mmm1p, Mdm10p, Mdm12p, Mdm34p, and a substoichiometric regulatory Miro GTPase Gem1p (Kornmann *et al*, 2009, 2011). Under conditions of *SCT1* overexpression, the ERMES mutants, *mmm1Δ*, *mdm10Δ*, *mdm12Δ*, *mdm34Δ*, and *gem1Δ*, showed an increase of at least 50% in SFA/UFA ratio compared to WT (Fig 5A), in line with a defect in mitochondrial lipid import. Accordingly, growth of ERMES mutants overexpressing *SCT1* was strongly retarded compared to WT and empty vector control (Fig 5B). In contrast, growth of a triple mutant devoid of 3 subunits of the EMC tethering ER to mitochondria (3x*emcΔ*, Lahiri *et al*, 2014) was not affected by *SCT1* overexpression, while that of the quadruple mutant (4x*emcΔ*, Lahiri *et al*, 2014) tested was slightly impaired (Fig 5B). Since the 3x*emcΔ* and 4x*emcΔ* mutants overexpressing Sct1p (Appendix Fig S7A) did not show increased accumulation of SFA (Fig 5A), this was probably not due to defective PS transfer.

Mitochondria are tethered to the vacuole by vCLAMP consisting of Ypt7p, Vps39p, and Tom40p, and by a second, independent OCS formed by Vps13p and Mcp1p (González Montoro *et al*, 2018). The SFA/UFA ratio of *ypt7Δ* and *vps39Δ* exhibited an increase around 30%, while that of *vps13Δ* did not significantly differ from WT (Fig 5A, Appendix Table S1). vCLAMP mutant *vps39Δ* showed delayed growth upon overexpressing *SCT1*, whereas growth of *ypt7Δ* and *vps13Δ* was only slightly affected (Fig 5B).

To exclude that the phenotypes induced by overexpressing *SCT1* in ERMES and vCLAMP mutants resulted from indirect effects related to Psd1p activity or to the level of *SCT1* overexpression, the following controls were done. Western blot analysis showed that the expression level of Psd1p was not decreased, and the level of Sct1p overexpression not increased in the tether mutants versus the WT (Appendix Fig S7A). Moreover, episomal expression of *PSD1* from a *PGK1*‐promoter, while restoring growth of the *psd1Δ* strain, did not rescue the growth phenotype of the ERMES and vCLAMP mutants under conditions of *SCT1* overexpression (Appendix Fig S7B).

Since lipid transport confers molecular species selectivity to PS‐to‐PE conversion (Fig 3), we next examined the effect of inactivating ERMES or vCLAMP on the molecular species selectivity of PS decarboxylation. The mutants *gem1Δ*, *mdm34Δ*, and *vps39Δ*, and the corresponding WT BY4741 were pulsed for 20 min with ^13^C_3_^15^N‐serine, and the labeled PS and PE molecular species synthesized were analyzed by high‐resolution mass spectrometry (Dataset EV3). Both ERMES mutants revealed a decrease in relative rate of *PS‐to‐*PE conversion compared to WT (Fig 5C), in agreement with impaired mitochondrial PS import (Kornmann *et al*, 2009, 2011). In contrast, the efficiency of *PS‐to‐*PE conversion in *vps39Δ* was not reduced, in agreement with previous observations (Elbaz‐Alon *et al*, 2014). These results indicate that under the conditions used, ER‐mitochondrial contacts are the main route of mitochondrial lipid import. Notably, the preference for decarboxylating *PS 32:2 and *PS 34:2 observed in WT was retained in the mutants (Fig 5D and E), yet the total production of di‐unsaturated *PE was markedly reduced in the ERMES mutants (Fig 5E). The species selectivity of *PE synthesis being retained suggests that Psd1p remains the main source of *PE in the ERMES mutants.

Taken together, the results show that the species selectivity of *PE synthesis is preserved in ERMES mutants and *vps39Δ*, suggesting that the lipid phenotypes of the tether mutants overexpressing *SCT1* (Fig 5A) are due to a decreased rate of mitochondrial lipid import.

### Rescue of ERMES mutants and *vps39Δ* overexpressing *SCT1* by exogenous ethanolamine and choline

**Figure 6:**
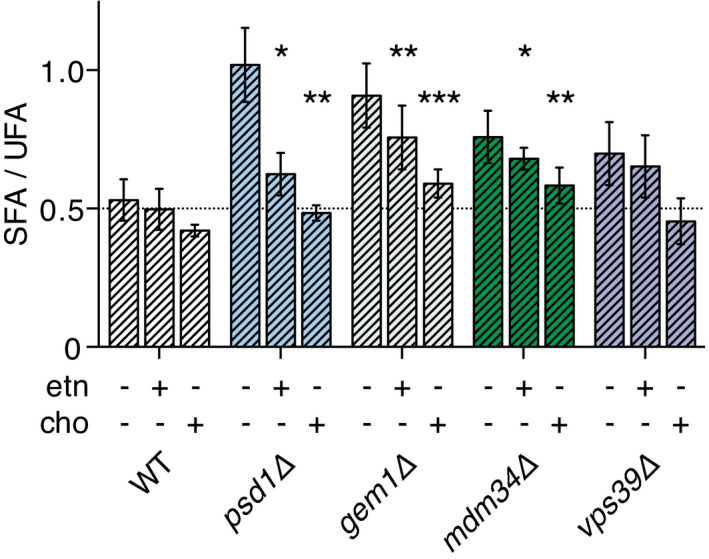
Effect of exogenous ethanolamine and choline on the accumulation of saturated acyl chains in ERMES mutants and *vps39Δ* SFA/UFA ratio of WT and indicated mutant strains overexpressing *SCT1* cultured without (data taken from Fig 5A) and with 1 mM ethanolamine (etn) or 1 mM choline (cho) as indicated. Data are presented as average ± SD (*n* ≥ 3). *P < 0.05, **P < 0.01, ***P < 0.001, unpaired *t*‐test of the indicated bar compared to the corresponding condition of WT. Underlying data for this figure can be found in Appendix Table S1.

Under the growth conditions used, *i.e*., culture media devoid of choline and ethanolamine, cells depend to a large extent on mitochondrial PSD activity for net synthesis of PE and PC. Supplementation of ethanolamine and choline reduces the cellular requirement for mitochondrial lipid synthesis by diverting lipid flux into the CDP‐ethanolamine and CDP‐choline branches of the Kennedy pathway, respectively (Fig 1A, Dimmer & Rapaport, 2017). Since both routes draw on the pool of unsaturated acyl‐CoA’s via PA and diacylglycerol (DAG, Boumann *et al*, 2004), choline and ethanolamine were expected to alleviate the putative feedback inhibition of Ole1p. In *psd1Δ* overexpressing *SCT1*, choline restored the SFA/UFA ratio to WT level (Fig 6). Ethanolamine did not completely reverse the phenotype (Fig 6), indicating that the demand for acyl‐CoA by flux through the CDP‐ethanolamine pathway was insufficient to compensate for the reduced mitochondrial UFA sink. Compared to *psd1Δ*, ethanolamine only partially (if at all) decreased the accumulation of SFAs conferred by *SCT1* overexpression in the ERMES mutants and *vps39Δ* (Fig 6). Choline strongly suppressed SFA accumulation in all strains, with the SFA/UFA ratio in *vps39Δ* returning to WT and in *mdm34Δ* and *gem1Δ* staying slightly above (Fig 6). The extents to which ethanolamine and choline reverse the slow growth phenotype of the *SCT1*‐overexpressing mutants (Appendix Fig S8) paralleled the corresponding decreases in SFA/UFA ratio. The incomplete restoration of the SFA/UFA ratios by the supplements in the tether mutants suggests that ERMES may facilitate PE and PC transfer and that Vps39p may mediate transfer of PE.

## Discussion

Biosynthesis of phospholipids in mitochondria is dependent on import of phospholipid precursors; however, the molecular details of mitochondrial lipid import remain elusive. Stable isotope labeling combined with MS analysis revealed that shorter, di‐unsaturated PS molecular species are preferentially converted to PE by mitochondrial Psd1p (Figs 2B, 3B, 4A and 5D). Since ER‐localized Psd1p does not distinguish between PS molecular species (Fig 3B and C), the transfer of PS from ER to mitochondria confers the molecular species selectivity. The preferred decarboxylation of shorter and more unsaturated PS molecular species in mammalian cells was previously interpreted in terms of the rate of transfer to mitochondria being inversely proportional to the molecular hydrophobicity of the PS molecular species, which led to the hypothesis that the efflux from the ER membrane constitutes the rate‐limiting step (Heikinheimo & Somerharju, 2002; Kainu *et al*, 2013). Taking advantage of *SCT1* overexpression to manipulate the acyl chain composition in yeast, this hypothesis was experimentally tested *in vivo*. The reduced synthesis of PE under conditions of *SCT1* overexpression (De Smet *et al*, 2012) and the aggravation of the *SCT1* overexpression phenotype in ERMES and vCLAMP deletion mutants provide the first *in vivo* evidence for lipid efflux from the donor membrane being rate‐limiting in intermembrane lipid transport.

To limit UFA availability and study its effect on lipid transfer to mitochondria, an artificial metabolic sink for SFA was introduced in yeast by overexpressing *SCT1*. The rise in SFA/UFA resulting from overexpressed Sct1p outcompeting Ole1p serves as a sensitive read‐out of interference with the mitochondrial UFA sink, as validated in *psd1Δ* and *crd1Δ*, and demonstrated in ERMES mutants. The rise in SFA/UFA is tentatively attributed to feedback inhibition of Ole1p caused by a reduced demand for unsaturated lipids, stimulating the incorporation of SFA into lipids by overexpressed Sct1p. In addition to mass action, product inhibition of lipid biosynthetic enzymes as demonstrated for PS synthase (Kannan *et al*, 2017) may contribute to this feedback mechanism.

The roles of ERMES and vCLAMP in mitochondrial lipid import are well‐established (reviewed in e.g., Dimmer & Rapaport, 2017). However, loss of either ERMES or vCLAMP hardly shows a lipid phenotype (Nguyen *et al*, 2012; Voss *et al*, 2012), probably due to the redundancy of mitochondrial lipid import pathways (Elbaz‐Alon *et al*, 2014). Pulsing with stable isotope‐labeled serine revealed a reduction in PS‐to‐PE conversion in ERMES mutants, in agreement with the delay in the conversion of PS to PC reported previously (Kornmann *et al*, 2009), whereas inactivation of vCLAMP in *vps39Δ* had little effect (Fig 5C). Limiting the availability of unsaturated lipids by overexpression of *SCT1* allows for a more sensitive read‐out of the effects on mitochondrial lipid import resulting from loss of ERMES or vCLAMP subunits. Based on a comparison of SFA/UFA ratios (Fig 5A), using *psd1Δ* (with a strongly reduced mitochondrial sink for unsaturated phospholipids) as a reference, ERMES appears to be the main contributor to mitochondrial import of lipids, PS first and foremost under the conditions used. EMC consists of multiple subunits and tethers to the TOM complex in the mitochondrial outer membrane. Deletion of multiple EMC genes is synthetically lethal with ERMES mutations and slows down PS‐to‐PE conversion (Lahiri *et al*, 2014). However, when judged by the criterion of SFA/UFA ratio under conditions of *SCT1* overexpression, EMC plays a minor role, if any, in PS import. Instead, the role of EMC in membrane protein biogenesis (Chitwood & Hegde, 2019) may account for the reported aberrant lipid metabolism of EMC mutants.

Even though the efficiency of decarboxylation of newly synthesized PS was not affected in a *vps39Δ* mutant (Fig 5C), deletion of *VPS39* and *YPT7* was found to enhance the *SCT1* overexpression phenotype (Fig 5A), suggesting that lipid transfer from the vacuole does contribute to the mitochondrial sink for di‐unsaturated phospholipids in wild‐type cells. Since Vps39p and Ypt7p are part of both vCLAMP and the HOPS complex that functions upstream supplying lipids to the vacuole by vesicular transport (Balderhaar & Ungermann, 2013; González Montoro *et al*, 2018), the present data do not allow distinction between defective HOPS or vCLAMP exacerbating the *SCT1* overexpression phenotype. The lack of rescue by ethanolamine of *vps39Δ* overexpressing *SCT1* (Fig 6) is in agreement with the recently identified third function of Vps39p, trafficking ethanolamine‐derived PE from ER to mitochondria independent of vCLAMP or HOPS (Iadarola *et al*, 2020). Inactivation of Vps13p did not affect the SFA/UFA ratio, consistent with its function in mitochondrial lipid import only becoming apparent in the absence of functional ERMES (Lang *et al*, 2015; John Peter *et al*, 2017; González Montoro *et al*, 2018). The defects in intermembrane transport caused by deficiency of ERMES or Vps39p under conditions of limiting UFA are not restricted to PS, but probably also pertain to PE and PC, as evidenced by the SFA/UFA ratios not returning to WT level when ethanolamine or choline is supplemented (Fig 6).

**Figure 7:**
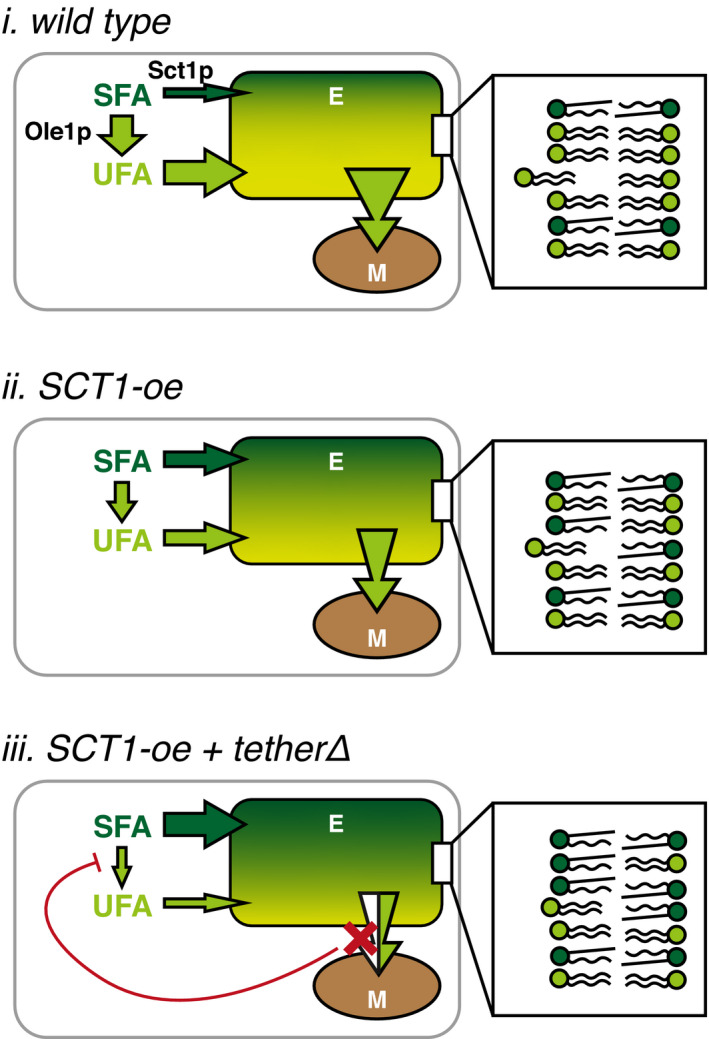
Model of the cross‐talk between the SFA sink introduced by *SCT1* overexpression and the mitochondrial UFA sink in wild‐type yeast and mutants disturbed in ERMES or vCLAMP Schematic representation of the relative contributions of Sct1p and Ole1p to the SFA (dark green)/UFA (light green) ratio, and its consequences for lipid flux from the endomembrane system (E) into mitochondria (M) in WT (i), WT overexpressing SCT1 (*SCT1‐*oe) (ii), and cells lacking a tether that overexpress *SCT1* (*SCT1‐*oe *tetherΔ*) (iii). In the latter, the flux of SFA into the pool of membrane lipids is further enhanced by the depicted feedback mechanism in response to partially obstructing (red cross) lipid entry into mitochondria. The insets show the tendency of a di‐unsaturated lipid to efflux from the endomembrane system for each condition, depicted as the extent of displacement from the bilayer.

Model membrane studies have shown that the rate of desorption of a lipid from a bilayer is determined by the lipid’s molecular structure and the physical properties of the donor membrane (reviewed in Somerharju, 2015). Our results provide evidence for molecular species‐selective lipid flow into mitochondria at OCS that is rate‐limited by lipid release from the donor membrane and facilitated in an additive manner by the lipid species’ concentration gradient between donor and acceptor membrane and by the presence of tethers that in the simplest model serve as passive conduits increasing the probability of transfer to the juxtaposed membrane (Fig 7). After the rate‐limiting step of lipid desorption from the ER membrane, the tethering complexes “catch” the lipids by providing a hydrophobic environment, shielding the lipids from the unfavorable, aqueous environment. This allows for lipid diffusion to the receiving (mitochondrial) membrane, according to the lipid concentration gradient.

Since desorption favors di‐unsaturated hydrophilic lipid species (Somerharju, 2015; Richens *et al*, 2017), their concentration gradient is of prime importance. In case of PS, the gradient is maintained by the enrichment of PS synthesis in MAM (Gaigg *et al*, 1995), where local biosynthesis of PS at OCS has been shown to drive lipid transport (Kannan *et al*, 2017), and by the efficient conversion of di‐unsaturated PS to PE by mitochondrial Psd1p upon transfer. Lowering the donor membrane’s UFA content by overexpression of *SCT1* reduces the steepness of the chemical gradient of di‐unsaturated PS, as well as its rate of desorption by decreasing membrane fluidity (Fig 7, *i* and *ii*). As a result, yeast cells overexpressing *SCT1* produce reduced proportions of newly synthesized di‐unsaturated PE and exhibit an overall reduced PE level (De Smet *et al*, 2012). Interference with the tethering complexes exacerbates the decrease in UFA content, most likely by causing the putative feedback inhibition of Ole1p activity that enables overexpressed Sct1p to sequester even more SFA in glycerolipids (Fig 7, *iii*). This model is in agreement with observations suggesting that tethering complexes lack lipid substrate specificity (AhYoung *et al*, 2015; Kawano *et al*, 2018), suggesting they serve as passive transfer “hubs” between membranes.

ERMES subunits are equipped with synaptotagmin‐like mitochondrial lipid‐binding protein (SMP) domains that facilitate lipid transfer by providing hydrophobic binding pockets for the released lipid (AhYoung *et al*, 2015; Kawano *et al*, 2018). In agreement with lipid release from the donor membrane being rate‐limiting for transfer, the endogenous lipids co‐purifying with overexpressed Mdm12p are enriched in di‐unsaturated, short 32:2 PC (AhYoung *et al*, 2015). Concentration gradient‐driven lipid transfer between membranes as proposed here is fully compatible with the hydrophobic channel structure of Vps13 (Li *et al*, 2020).

An early link between cellular UFA content and mitochondrial function was the identification of the *mdm2* mutation that confers defective intracellular mitochondrial movement, as an *OLE1* allele (Stewart & Yaffe, 1991). In support of UFA availability limiting mitochondrial lipid import, the loss of fitness induced by deletion of *MDM34* was compensated by a point mutation in *MGA2* that confers twofold upregulation of the *OLE1* transcript, and by supplementation with oleic acid (Szamecz *et al*, 2014). Accordingly, the growth phenotype of ERMES mutants was alleviated by overexpression of *OLE1* (Kojima *et al*, 2016).

Mitochondrial function requires the non‐bilayer lipids PE and CL (Basu Ball *et al*, 2018), and their non‐bilayer propensity critically depends on UFA content (Renne & de Kroon, 2018). The proposed concentration gradient‐driven transfer of preferentially di‐unsaturated PS and PA from ER to mitochondria by tether‐facilitated diffusion warrants optimal PE and CL molecular species composition (*cf*. Calzada *et al*, 2019), explaining why PE and CL synthesis have been retained in mitochondria during evolution. The presence of multiple tethers connecting mitochondria to the endomembrane system, some with redundant functions in lipid transport, underscores the importance of this process.

Inactivation of Crd1p increases lipid saturation under *SCT1* overexpression, similar to loss of Psd1p (Fig 4C), indicating that mitochondria also serve as a sink for unsaturated molecular species of the CL precursor PA. In the presence of choline, SFA/UFA ratios of the ERMES‐mutants overexpressing *SCT1* are not completely restored to wild‐type level (Fig 6), suggesting some preference for transport of unsaturated PC species into mitochondria. Schneiter *et al* (1999) observed only a slight enrichment of unsaturated PC species in purified mitochondria compared to microsomes, whereas in mitochondrial PI no enrichment of unsaturated species was detected. Accordingly, on non‐fermentable carbon source (glycerol), the unsaturation of PE and CL species is increased, whereas this is not apparent for PC and PI (Klose *et al*, 2012). To explain these observations, we propose that the rates of net PC and PI transfer into mitochondria are slower than for PS and PA, because PC and PI are not quantitatively metabolized in mitochondria and therefore do not experience a steep concentration gradient between ER and mitochondria. As a consequence, di‐unsaturated molecular species would lose their competitive edge, *i.e*., differences in rate of efflux of lipid species from the donor membrane have less or no impact on the kinetics of net transfer of PC and PI.

The results presented have implications for our understanding of cellular membrane lipid homeostasis. The identification of mitochondria as a sink for phospholipids with unsaturated acyl chains accounts for the rise in unsaturated over saturated acyl chain content in yeast cells cultured on non‐fermentable versus fermentable carbon source (Tuller *et al*, 1999). The molecular species‐selective PS transfer to mitochondria explains the longstanding observation that newly synthesized PS is more readily converted to PE than pre‐existing PS (Vance, 1991; Gaigg *et al*, 1995). Moreover, it accounts for the higher SFA content of PS compared to PE and PC (Wagner & Paltauf, 1994; Schneiter *et al*, 1999; Bergenholm *et al*, 2018). The continuous equilibration of newly synthesized PC and its di‐ and mono‐methylated precursors between ER and mitochondria reported previously (de Kroon *et al*, 2003) further supports the notion of gradient‐driven lipid transfer at membrane contact sites.

In conclusion, pulse labeling with stable isotope‐labeled serine and the dynamic manipulation of lipid acyl chain composition by overexpressing Sct1p have uncovered mechanistic details of mitochondrial lipid import and identified the mitochondrial molecular species selectivity filter as a novel player in membrane lipid homeostasis. These results provide new insights in how cells establish organelle specific lipidomes.

## Materials and Methods

### Strains, plasmids, and culture conditions

Yeast strains used were derived from W303 and BY4741 and are listed in Appendix Table S2. Strains were maintained and pre‐cultured in synthetic glucose medium (SD), containing per liter 6.7 g of yeast nitrogen base (Difco, B/D Bioscience), 20 g of glucose, 20 mg of adenine, 20 mg of arginine, 20 mg of histidine, 60 mg of leucine, 230 mg of lysine, 20 mg of methionine, 300 mg of threonine, 20 mg of tryptophan, and 20 mg uracil. Strains containing plasmids listed in Appendix Table S2 were cultured in drop‐out media devoid of the appropriate components.

The *PSD1*‐expression vector was constructed from pYES2‐*PSD1*‐HA by releasing the *PSD1‐HA* fragment by double digestion and subsequent ligation into pYPGK18. Correct insertion was verified by restriction analysis, and Psd1p expression was verified by complementation of the *psd1Δ* mutant.

Overnight cultures were diluted in SD and cultured to exponential growth. Cells were harvested, washed with water, and inoculated at OD_600_ < 0.05 in pre‐warmed synthetic galactose medium (SGal), and cultured overnight. SGal contained per liter 6.7 g in‐house mixed inositol‐ and choline‐free YNB (Griac *et al*, 1996; Renne *et al*, 2020), 20 g of galactose (Acros Organics), 13.5 mg *myo‐*inositol (final concentration 75 µM), and amino acids as above, with or without 0.5 g glucose as indicated. Strains were cultured at 30°C while shaking (200 rpm) and harvested during exponential growth (OD_600_ 0.3–0.8). Cell pellets were stored at −20°C until further processing.

Growth phenotypes were determined using a serial dilution spot assay. Cells were grown overnight in SD, diluted, and cultured to exponential growth (OD_600_ 0.5–1.0). A culture volume corresponding to 0.5 OD_600_ units was harvested, washed with water, resuspended in water at OD_600_ of 1, and serially diluted in 10‐fold increments to 10^−4^. 4 µl aliquots of each dilution were spotted on SGal‐agar plates and incubated at 30°C for the number of days indicated.

### ^13^C_3_^15^N‐serine pulse labeling and lipid analysis by shotgun lipidomics

Yeast strains were cultured to early exponential growth (OD_600_ 0.4–0.6) in 100 ml SGal or SGal ‐ura +0.05% glucose medium as above. ^13^C_3_^15^N‐serine (Cambridge Isotope Laboratories) was added to a final concentration of 100 mg/l. After 20 min, cellular metabolism was quenched by the addition of perchloric acid (70%, Sigma‐Aldrich) to a final concentration of 0.66 M and rapid cooling in an ice‐water bath. Cells were pelleted, washed 3× with 155 mM ammonium formate, snap‐frozen in liquid nitrogen, and stored at −20°C until further processing. Lipids were extracted using a two‐step extraction procedure (Ejsing *et al*, 2009) and analyzed by shotgun lipidomics (Ejsing *et al*, 2009; Casanovas *et al*, 2014). Briefly, cells were dispersed at 5 OD_600_ units per ml in 155 mM ammonium formate and lysed using glass beads. Lipids were extracted from cell lysates corresponding to 0.4 OD_600_ units in 200 µl buffer that were spiked with a cocktail of lipid standards. 990 µl chloroform/methanol (17:1, v/v) was added followed by vigorous shaking for 120 min at 4°C. The lower organic phase was collected after centrifugation. The remaining aqueous phase was re‐extracted with 990 µl chloroform/methanol (2:1, v/v) for 60 min, and the lower organic phase was collected. Lipid extracts were vacuum dried and dissolved in chloroform/methanol 2:1 (v/v). Lipid extracts were analyzed in negative and positive mode on an Orbitrap Fusion Tribrid mass spectrometer (Thermo Fisher Scientific) equipped with a Triversa Nanomate (Advion Biosciences) (Almeida *et al*, 2015). Lipidomics data analysis was performed using ALEX^123^ software (Husen *et al*, 2013; Pauling *et al*, 2017).

### ^2^H_3_‐serine pulse labeling and lipid analysis by tandem MS

Wild‐type yeast harboring pYES2‐*SCT1*‐HH or pYES2‐Empty was cultured to early exponential growth (OD_600_ 0.4–0.6) in 100 ml SGal ‐ura medium as above. At time zero deuterium‐labeled, ^2^H_3_‐serine (Cambridge Isotope Laboratories) was added to a final concentration of 100 mg/l. After 20 min, cellular metabolism was quenched by addition of trichloroacetic acid to a final concentration of 5% (w/v) and rapid cooling on ice. Cells were pelleted, washed with water, and stored at −20°C until further processing. Lipids were extracted as detailed below, dried under nitrogen, and stored at −20°C. For ESI‐MS/MS analysis, lipid extracts were directly injected and analyzed on an API4000 triple quadrupole instrument (Applied Biosystems). Detection of PS and ^2^H_3_‐PS was in negative mode using neutral loss scans for 87 and 90 amu, respectively. PE and ^2^H_3_‐PE were detected in positive ion mode by neutral loss scans for 141 and 144 amu, respectively. PC was detected in the positive mode by parent ion scanning for *m/z* 184. Data were converted to mzML format and analyzed using XCMS version 1.52.0 (Smith *et al*, 2006) running under R version 3.4.3. All data were isotope corrected as described (Yang & Han, 2011).

### *In vitro* Psd1p assay

The *psd2Δ* strain was cultured in semi‐synthetic lactate medium (Daum *et al*, 1982) to late exponential growth (OD_600_ 2.5), harvested, and washed with 1 mM EDTA. Spheroplasts were prepared as described (Daum *et al*, 1982) using Zymolyase 100T (Seikagaku; 0.5 mg/g cells wet weight). The spheroplasts were resuspended in ice‐cold D‐buffer (0.6 M sorbitol, 10 mM Tris–HCl pH 7.4) containing protease inhibitors (complete protease inhibitor cocktail, Roche; 1 tablet per 50 ml), and lysed by 10 strokes in a Dounce homogenizer. Unbroken cells and nuclei were removed by two low‐spin centrifugation steps (1,400 *g* and 1,500 *g*, respectively, 4 min, 4°C). The mitochondrial fraction was pelleted (9,700 *g*, 10 min, 4°C), resuspended in assay buffer (0.6 M sorbitol, 1 mM EDTA, 50 mM Tris–HCl pH 7.4), and stored at −80°C. Protein concentration was determined using the BCA assay (Pierce) in the presence of 0.1% SDS with BSA as standard.

*In vitro* PS decarboxylation was analyzed at 0.5 mg/ml total mitochondrial protein in assay buffer also containing 0.1% (w/v) Triton X‐100. Synthetic PS molecular species (Avanti Polar Lipids) were added at 50 µM from 10x stocks in assay buffer containing 0.1% Triton X‐100. The reaction mixture was incubated at 30°C (with vigorous shaking) and 100 µl samples were drawn at *t* = 0 and *t* = 1 h, and the reaction was stopped by adding these samples to chloroform/methanol (1:1) and rapid cooling on ice. Lipids were extracted, dried under nitrogen, and stored at −20°C.

Lipid extracts were analyzed for PS and PE by LC‐MS. Chromatography was performed on a hydrophilic interaction liquid chromatography (HILIC) column (2.6 µm HILIC 100 Å, 50 × 4.6 mm, Phenomenex, Torrance, CA), by elution with a gradient from acetonitrile/acetone (9:1, v/v) to acetonitrile/water (7:3 v/v, containing 10 mM ammonium formate), both containing 0.1% (v/v) formic acid, at a flow rate of 1 ml/min. The column outlet of the LC was connected to a heated electrospray ionization (hESI) source of an Orbitrap Fusion Tribrid mass spectrometer (Thermo Fisher Scientific). Full spectra were collected from *m/z* 400 to 1,150 at a resolution of 120,000 in negative mode. Data were converted to mzML format and analyzed as above (Smith *et al*, 2006; Yang & Han, 2011).

### Lipid extraction

Cell pellets were resuspended in 600 µl water, transferred to cooled 2‐ml Eppendorf tubes containing 0.5 ml of acid washed glass beads (Sigma), and bead‐bashed for 5 min at RT (Qiagen TissueLyser II). Lipid extracts were prepared using a modified version of the Bligh and Dyer lipid extraction (Bligh & Dyer, 1959). Briefly, the 600 µl cell lysate was transferred to a glass tube containing 1,360 µl methanol and 620 µl chloroform, and 20 µl 1 M HCl was added. The mixture was vortexed briefly and kept on ice for 2 min. Next, 600 µl chloroform and 600 µl 0.1 M HCl were added, and the mixture was vortexed briefly, kept on ice for 2 min, and centrifuged for 4 min at 3,000 *g* at 4°C to induce phase separation. The organic phase was collected. After adding 120 µl 1 M KCl to the water phase, it was washed with 600 µl chloroform, and the organic phases were pooled. The combined organic phase was washed with 0.1 M KCl, and after adding 120 µl isopropanol dried in a water bath (40°C) under N_2_‐flow. Phospholipid concentrations were determined according to Rouser *et al* (1970), after destruction in 70% perchloric acid for 1 h at 180°C.

Lipid extracts from the *in vitro* Psd1p assay samples were prepared as above using 6x smaller volumes.

### Analysis of acyl chain composition by GC‐FID

Total lipid extracts corresponding to 200 nmol phospholipid phosphorus were transesterified by heating for 2.5 h at 70°C in 2.5 ml 2.5% (v/v) methanolic H_2_SO_4_. Fatty acid methyl esters (FAME) were extracted by addition of 2.5 ml hexane and 2.5 ml water. After collecting the organic phase, the aqueous phase was washed with hexane, and the organic phases were pooled and washed with water until the pH of the water phase was neutral. 120 µl isopropanol was added, and the sample was dried in a water bath (40°C) under N_2_‐flow. The FAME were dissolved in 1 ml hexane, transferred to an Eppendorf tube, and centrifuged for 10 min (14,000 *g* at 4°C) to remove any residual particles. Next, 900 µl of the supernatant was concentrated to a volume of 50–100 µl under N_2_‐flow.

FAME were analyzed by GC‐FID using splitless injection (2 µl injection volume, inlet temperature 230°C, 1 min splitless time with a split flow of 20 ml/min) on a Trace*GC Ultra* (Thermo Scientific) equipped with a biscyanopropyl‐polysiloxane column (Restek), using N_2_ as carrier gas (1.3 ml/min, constant flow). After injection, the samples were concentrated on the column by keeping the temperature at 40°C for 1 min, after which the column was rapidly heated to 160°C (30°C/min). FAME were separated using a temperature gradient from 160°C to 220°C (4°C/min), and signal was detected using FID at 250°C (H_2_/air with N_2_ as make‐up).

Integrated peaks were identified and calibrated using a commercially available FAME standard (63‐B, Nu‐Chek‐Prep). Acyl chain compositions are presented as mol% of the four most abundant acyl chains (C16:0, C16:1, C18:0 and C18:1) that were recovered in all samples.

### Analysis of *OLE1* expression by RT–qPCR

A cell pellet corresponding to 20–25 OD_600_ units was lysed with glass beads, and RNA was isolated from the cell lysate using the RNeasy Mini Kit and RNase‐free DNase Set (both Qiagen) according to the manufacturer’s instructions. RNA quality and quantity were checked by agarose gel electrophoresis and spectrophotometry using a NanoDrop 2000 spectrophotometer (Thermo Fisher Scientific), respectively. cDNA was synthesized from 1 µg RNA using Superscript III Reverse Transcriptase (Invitrogen) and oligo (dT)_12‐18_ primers according to the manufacturer’s instructions. RT–qPCR was performed using TaqMan Universal PCR mastermix 2× (Applied Biosystems, Thermo Fisher Scientific) supplemented with AmpErase UNG (Applied Biosystems, Thermo Fisher Scientific), using commercially available TaqMan probes and primers for *OLE1* (Sc04122147_s1) and *ACT1* (Sc04120488_s1, Thermo Fisher Scientific). PCRs were run on a ViiA 7 Real Time PCR machine (Thermo Fisher Scientific), and data were analyzed using corresponding software. C_t_ values were averaged from two technical duplicates, and cDNA‐devoid reactions were included as negative control. Gene expression was analyzed as 2^−ΔΔCt^ (Livak & Schmittgen, 2001), and normalized to *ACT1* as housekeeping gene.

### Western Blot analysis

Proteins were extracted from cell pellets (corresponding to 1 OD_600_ unit) by NaOH treatment and boiling in sample buffer as described (Kushnirov, 2000). Equal amounts were loaded based on culture density (Appendix Fig S1B) or protein quantification (Appendix Fig S7A). For protein quantification, aliquots of each sample were acetone precipitated, followed by solubilization in urea/guanidine and protein determination using the Bradford assay as described (Sheen, 2016).

Proteins were analyzed using primary antibodies against Sct1p‐HA (anti‐HA, Thermo Scientific), Psd1p (kind gifts from Dr. Thomas Becker (Horvath *et al*, 2012), used in Appendix Fig S1B, and from Dr. Steven Claypool (Onguka *et al*, 2015), used in Appendix Fig S7A), Gpd1p (anti‐GAPDH, Thermo Fisher Scientific), and Dpm1p (Thermo Fisher Scientific). Proteins were visualized using horse radish peroxidase conjugated secondary antibodies (Bio‐Rad or Jackson Laboratories) or IRdye‐conjugated secondary antibodies (Li‐Cor Biosciences, Lincoln, NE).

### Statistical analysis

Acyl chain composition was analyzed using a multiple *t*‐test, analyzing each acyl chain percentage individually and using the False Discovery Rate approach (Q = 5%). SFA/UFA ratios were analyzed by unpaired, parametric *t*‐test. All statistical analyses were done using GraphPad Prism 6 software.

## Author contributions

MFR, CHDS, and AIPMdK conceived the study; MFR, XB, CSE, and AIPMdeK contributed to funding acquisition; MFR, XB, MWJH, ASB, MH, RRS, TAE, XM, RCC, JFB, and CHDS performed experiments; MFR, XB, JFB, CSE, and AIPMdK developed methodology; MFR and AIPMdK supervised the study; MFR and AIPMdK visualized data; MFR and AIPMdK wrote original draft; all authors reviewed and edited the manuscript.

## Conflict of interest

The authors declare that they have no conflict of interest.

## Supporting information



Appendix

Dataset EV1

Dataset EV2

Dataset EV3

## Data Availability

This study includes no data deposited in external repositories. Lipidomics data related to this study are provided as supplementary datasets (Datasets [Link], [Link], [Link]).
