# Supplementary material for: Molecular species selectivity of lipid transport creates a mitochondrial sink for di‐unsaturated phospholipids
Source: EMBO J. 2021 Dec 7;41(2):e106837. doi: 10.15252/embj.2020106837 (PMC8762554; doi:10.15252/embj.2020106837)
Supplement: Supplementary file 1 — Appendix [file EMBJ-41-e106837-s003.docx]

**Appendix**

**Molecular species selectivity of lipid transport**

**creates a mitochondrial sink for di-unsaturated lipids**

**Table of contents**

**Appendix Figure S1** Growth of Psd1-mutants on SD and SGal media, and Psd1-expression levels on SGal medium

**Appendix Figure S2** Molecular species profiles of PS, PE and PC of PSD mutants

**Appendix Figure S3** Non-selective conversion of PS molecular species by Psd1 *in vitro*

**Appendix Figure S4** Profiles of the C32 and C34 molecular species of PS, PE, and PC in wild type BY4741 under conditions of *SCT1*-overexpression *versus* control (pEmpty) as determined by ESI-MS/MS

**Appendix Figure S5** Growth of mitochondrial mutants on synthetic galactose medium is improved by addition of 0.05% glucose

**Appendix Figure S6** Proportion of di-unsaturated molecular species in newly synthesized PS and PE increases with Psd1 activity in mitochondria

**Appendix Figure S7** Psd1p and Sct1p expression levels are not affected in ERMES, vCLAMP and EMC mutants overexpressing *SCT1*, and episomal Psd1 expression does not rescue ERMES or vCLAMP mutants overexpressing *SCT1*

**Appendix Figure S8** Effect of ethanolamine and choline on the growth of WT and indicated mutants overexpressing *SCT1* (p*SCT1*) *vs.* control (pEmpty)

**Appendix Table S1** Acyl chain composition of WT and indicated mutants under *SCT1*-overexpression (p*SCT1*) *vs*. empty vector control (pEmpty)

**Appendix Table S2** Yeast strains and plasmids used in this study

**
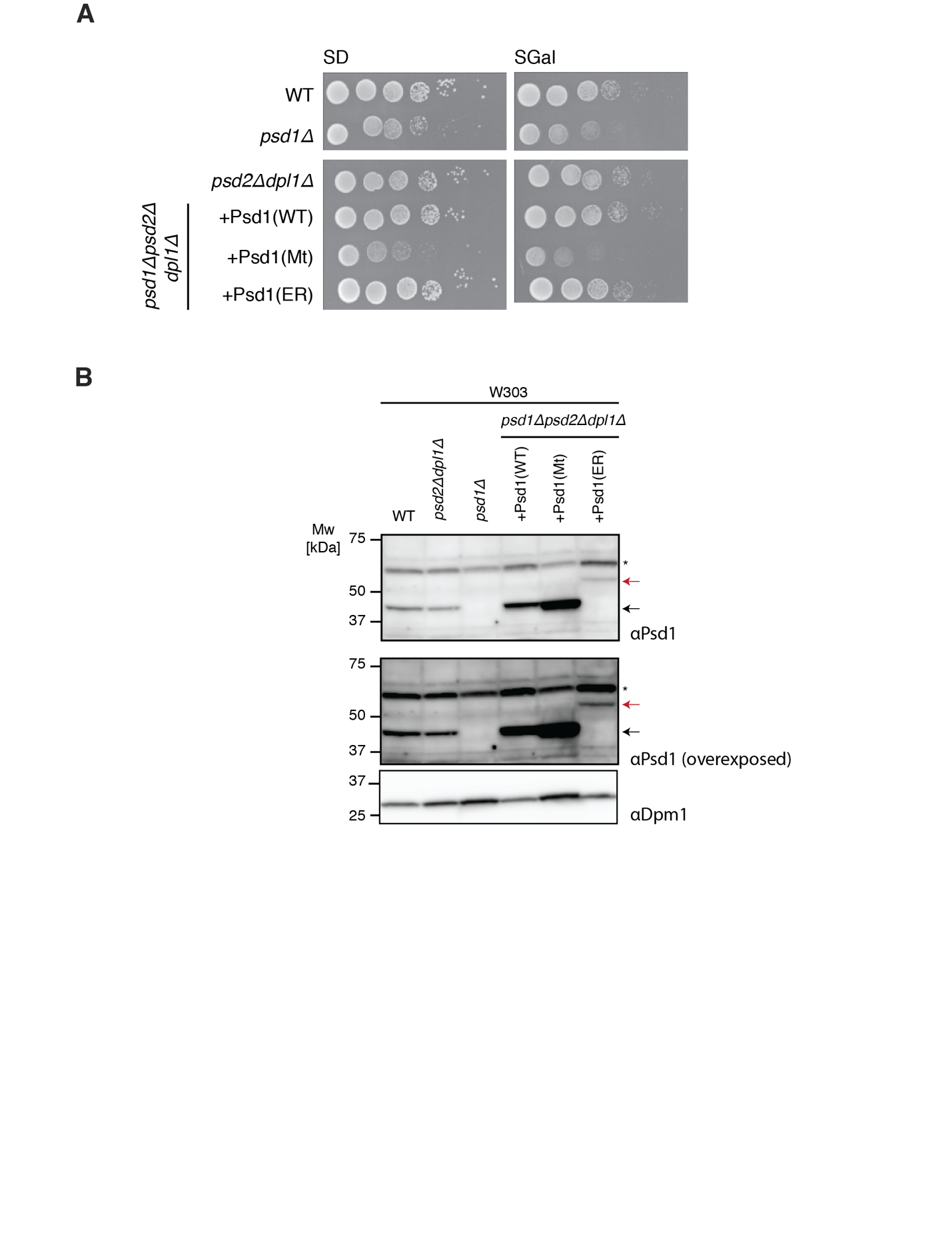
**

**Appendix Figure S1. Growth of Psd1-mutants on SD and SGal media, and Psd1-expression levels on SGal medium.**

(A) Serial dilutions (10^0^ – 10^-5^) of wild type and indicated mutant strains in W303 background were spotted on SD and SGal and incubated at 30°C for 3 days. (B) Western blot analysis of Psd1p expression in the indicated strains*.* Antibodies used were anti-Psd1p and anti-Dpm1p (loading control). Black arrow indicates WT Psd1p and Psd1(Mt); red arrow indicates Psd1(ER); * marks a non-specific band. Molecular weights (kDa) of the marker bands are indicated.

**
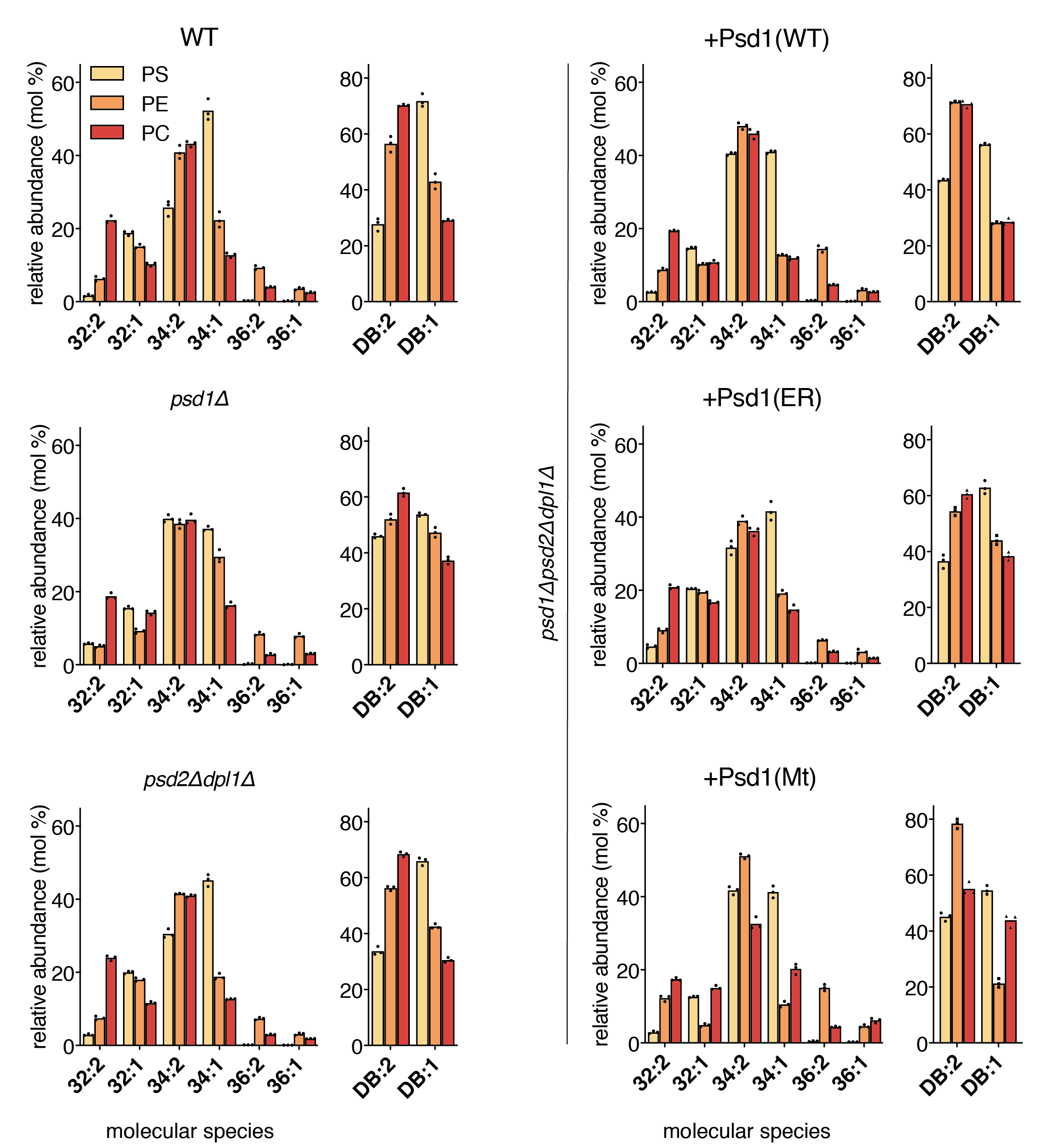
**

**Appendix Figure S2. Molecular species profiles of PS, PE and PC of PSD mutants.**

Molecular species profile (left) and total number of double bonds per phospholipid (right) of PS, PE, and PC are shown for the indicated mutant strains. Lipid extracts from the indicated strains prepared after the 20 min pulse (see Figures 2 and 3) were analyzed by shotgun lipidomics. Percentages of the molecular species representing at least 2% of PS, PE, or PC are included. The relatively high C36 content in PE is attributed to contribution of the isobaric phosphatidyldimethylethanolamine species. Data are presented as mean of 3 biological replicates, with the individual values indicated. Numerical data can be found in Dataset EV1.

**
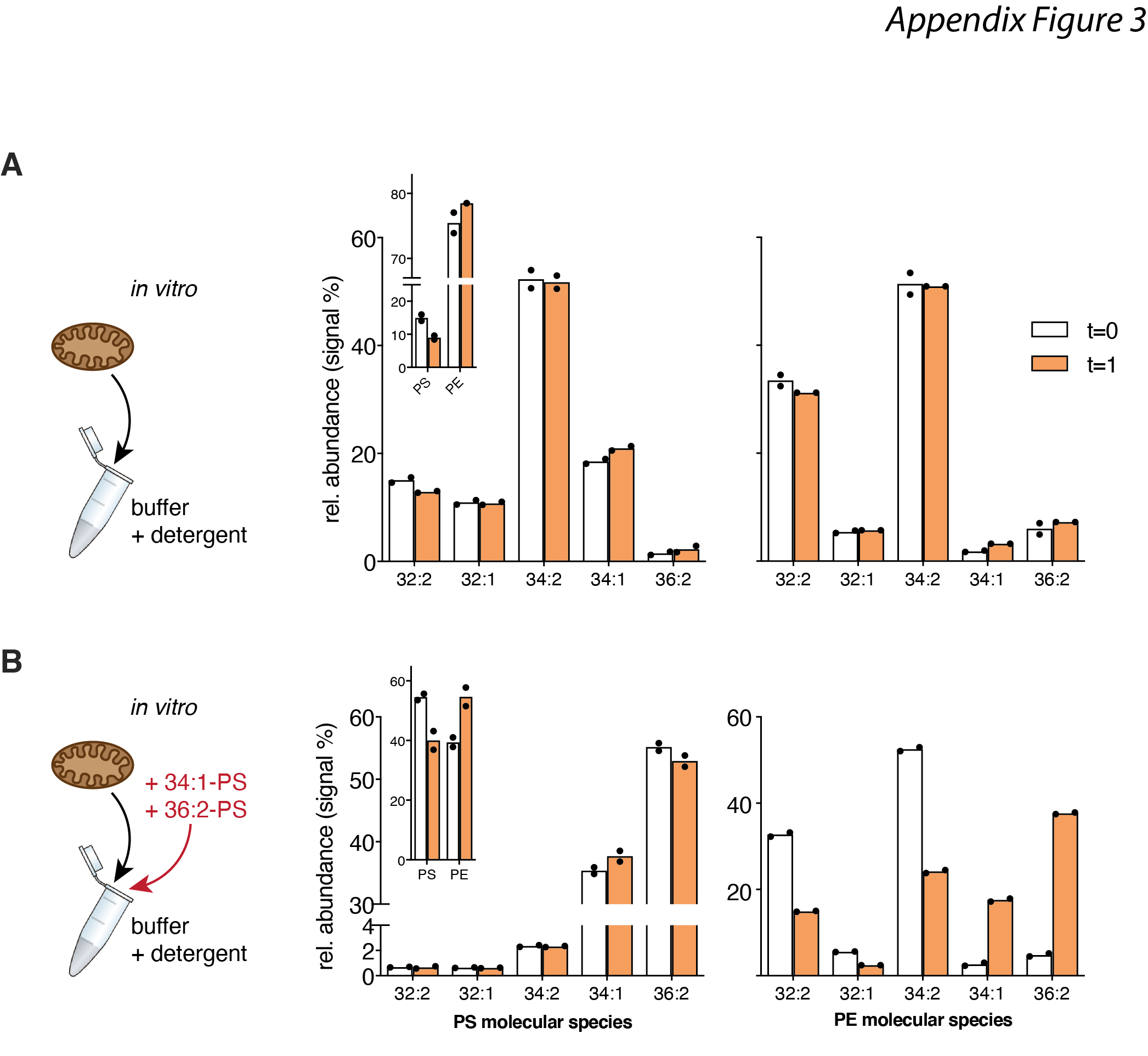
**

**Appendix Figure S3. Non-selective conversion of PS molecular species by Psd1 *in vitro.***

Molecular species profiles of PS and PE were recorded by LC-MS before and after 1 h of incubating detergent-solubilized mitochondria from a *psd2∆* strain at 30^o^C without (A) or with (B) the exogenous PS molecular species indicated. The insets comparing the summed signal of PS and PE species as percentage of the total (PS+PE+PI) signal at t=0 and t=1 h, show net conversion of PS to PE in the lower panel. As the PS species profiles are unaffected after 1 hour incubation (compared to t=0), the data shows that Psd1 has no molecular species preference *in vitro*. Bars represent the mean of two biological replicates with the individual values indicated. Relative abundance is shown for molecular species that contribute at least 1% of total PS or PE.


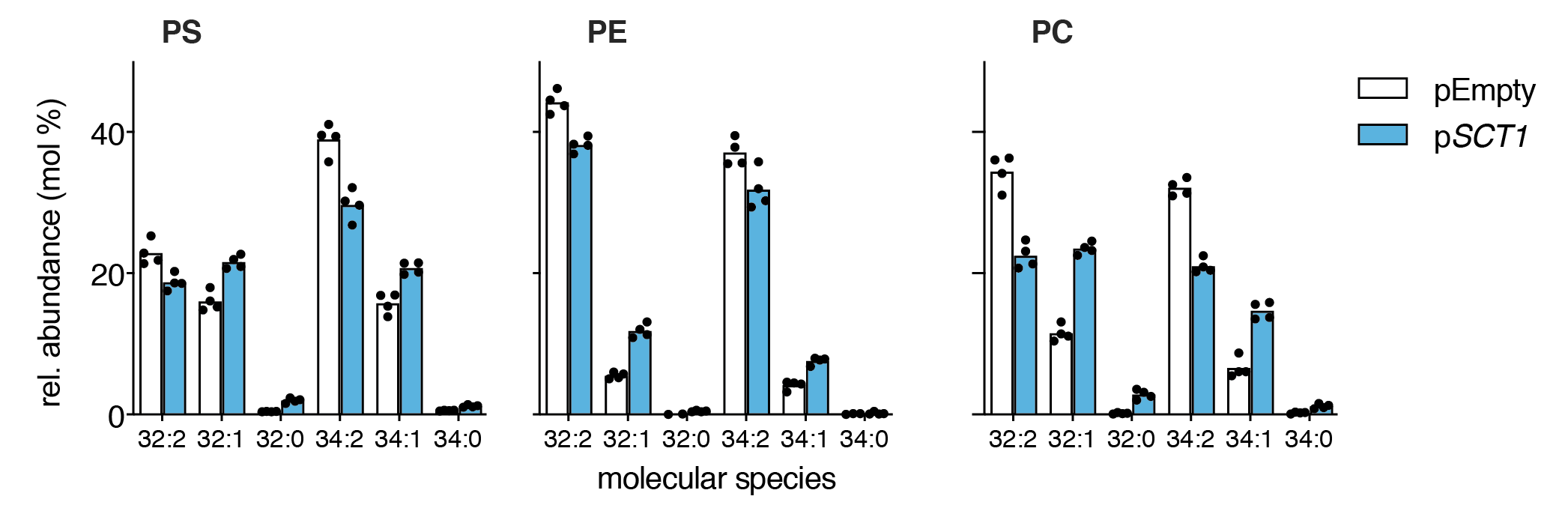


**Appendix Figure S4.** **Profiles of the C32 and C34** **molecular species of PS, PE, and PC in wild type BY4741 under conditions of *SCT1*-overexpression *versus* control (pEmpty) as determined by ESI-MS/MS.**

Bars represent the mean of four biological replicates with the individual values indicated. Numerical data can be found in Dataset EV2.


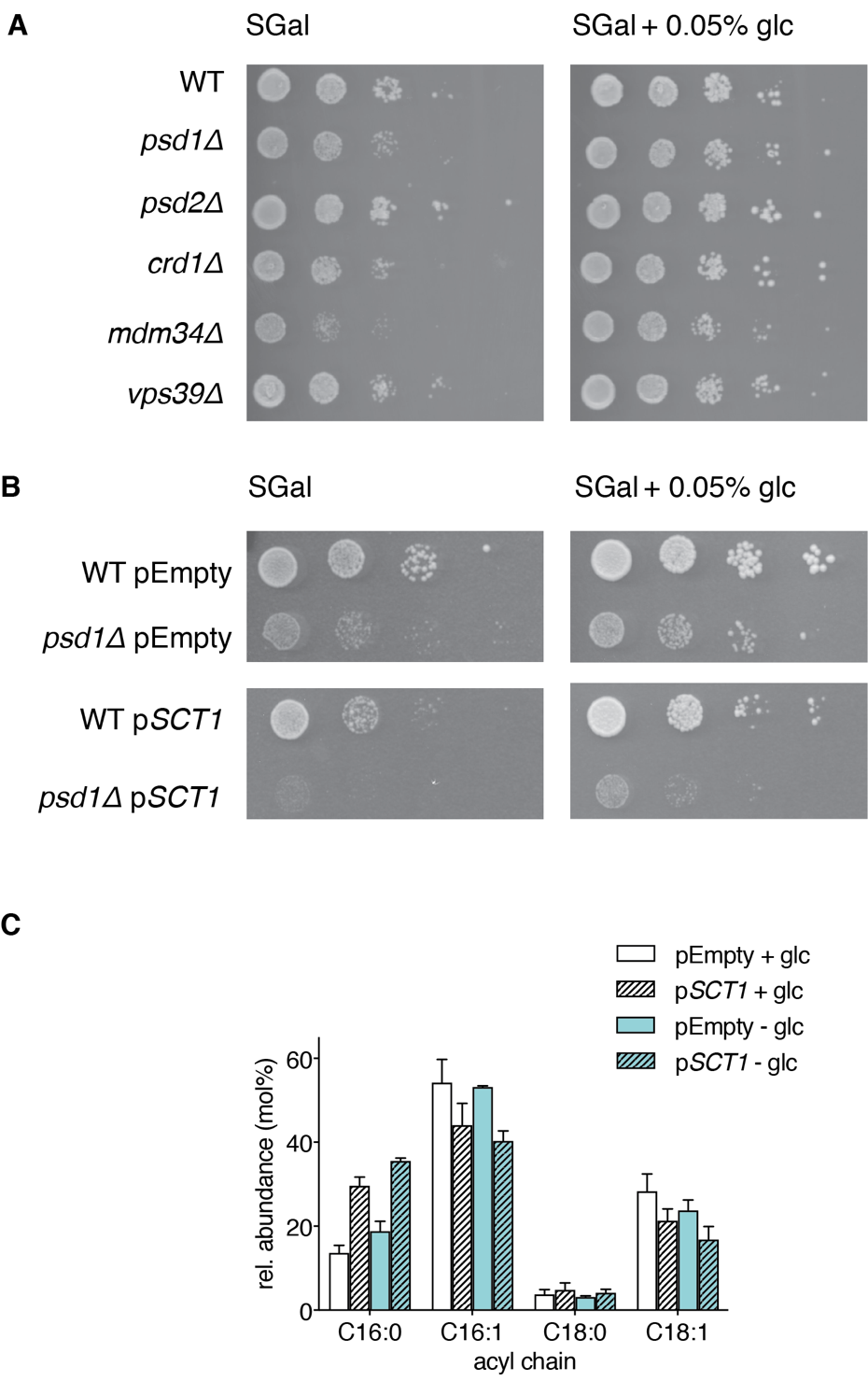


**Appendix Figure S5. Growth of mitochondrial mutants on synthetic galactose medium is improved by addition of 0.05% glucose.**

(A) Growth of WT, *psd2Δ,* and mutant strains disturbed in mitochondrial biogenesis on SGal (left) or SGal + 0.05% glucose (right). Serial dilutions (10^-1^ – 10^-5^) were spotted and incubated for 3 days at 30°C. (B) Growth of WT and *psd1Δ* overexpressing *SCT1* (p*SCT1*) *vs.* empty vector control (pEmpty) on SGal (left) or SGal + 0.05% glucose (right). Serial dilutions (10^-1^ – 10^-4^) were spotted and incubated for 3 days at 30°C. (C) Acyl chain composition of WT overexpressing *SCT1* (p*SCT1*; dashed bars) *vs.* empty vector control (pEmpty; open bars) cultured in SGal + 0.05% glucose (white bars, data from Fig 3B) or SGal (green bars). Data is depicted as mean + SD (n ≥ 3).


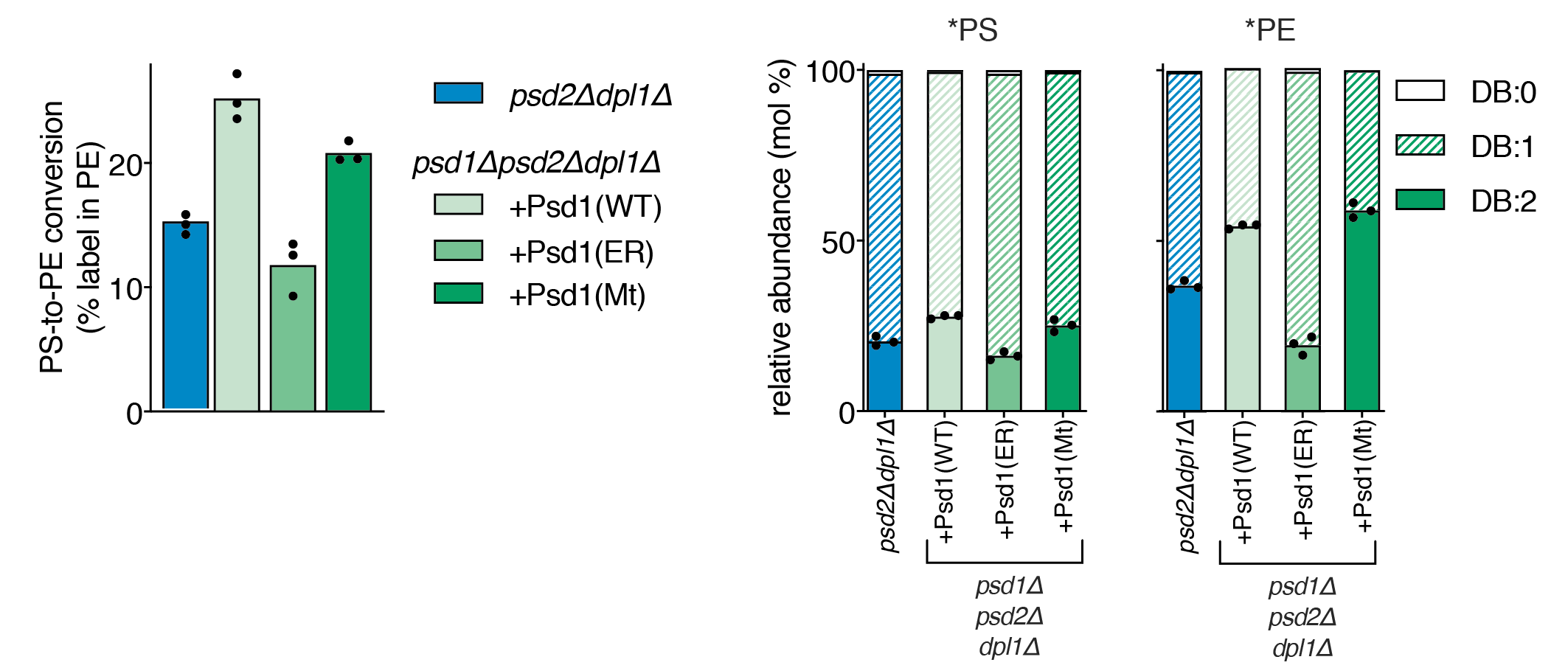


**Appendix Figure S6. Proportion of di-unsaturated molecular species in newly synthesized PS and PE increases with Psd1 activity in mitochondria.**

PS decarboxylase activity (data taken from Figs 2A and 3A) and proportions of di-unsaturated (DB:2), mono-unsaturated (DB:1) and di-saturated (DB:0) molecular species in ^13^C_3_^15^N -labeled PS (*PS) and ^13^C_2_^15^N -labeled PE (*PE) after 20 min incubation with ^13^C_3_^15^N-serine, of the W303 mutant strains indicated. Individual data (n=3) are derived from the experiment shown in Figures 2 and 3, and can also be found in Dataset EV1.


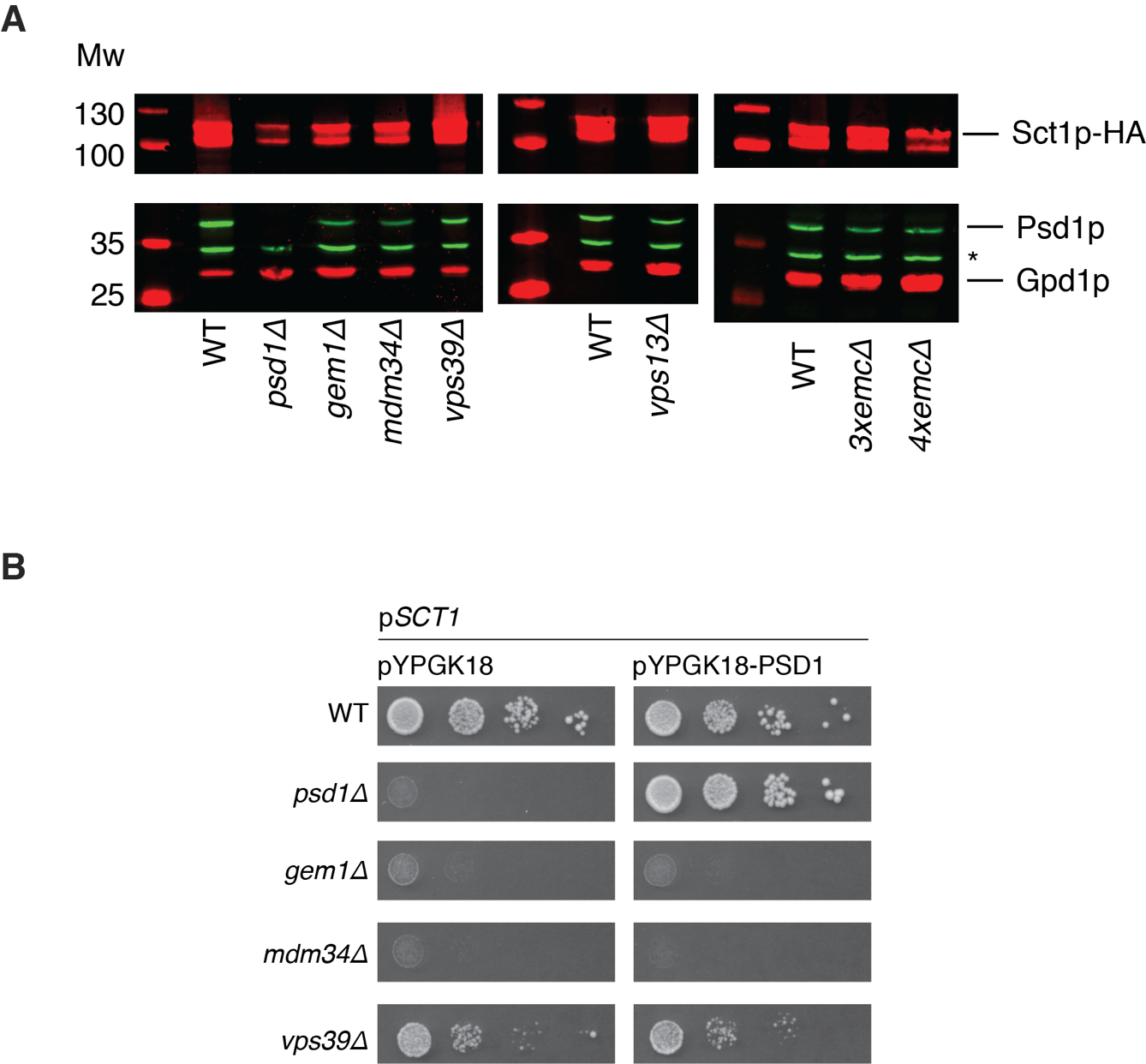


**Appendix Figure S7. Psd1p and Sct1p expression levels are not affected in ERMES, vCLAMP and EMC mutants overexpressing *SCT1,* and episomal Psd1 expression does not rescue ERMES or vCLAMP mutants overexpressing *SCT1*.**

(A) Western blot analysis of the (over)expression of Sct1p and Psd1p in the indicated strains overexpressing *SCT1.* Antibodies used were anti-HA (Sct1p-HA; red), anti-Psd1p (green) and anti-Gpd1p (loading control; red); * indicates a non-specific band. Molecular weights (kDa) of the marker bands are indicated.

(B) Growth of WT and indicated mutants overexpressing *SCT1* co-transformed with pYPGK18-*PSD1* expressing *PSD1* from the *PGK1* promoter or with the corresponding empty vector (pYPGK18). Serial dilutions (10^-1^ – 10^-4^) were spotted on SGal + 0.05% glucose plates and incubated for 4 days at 30°C.


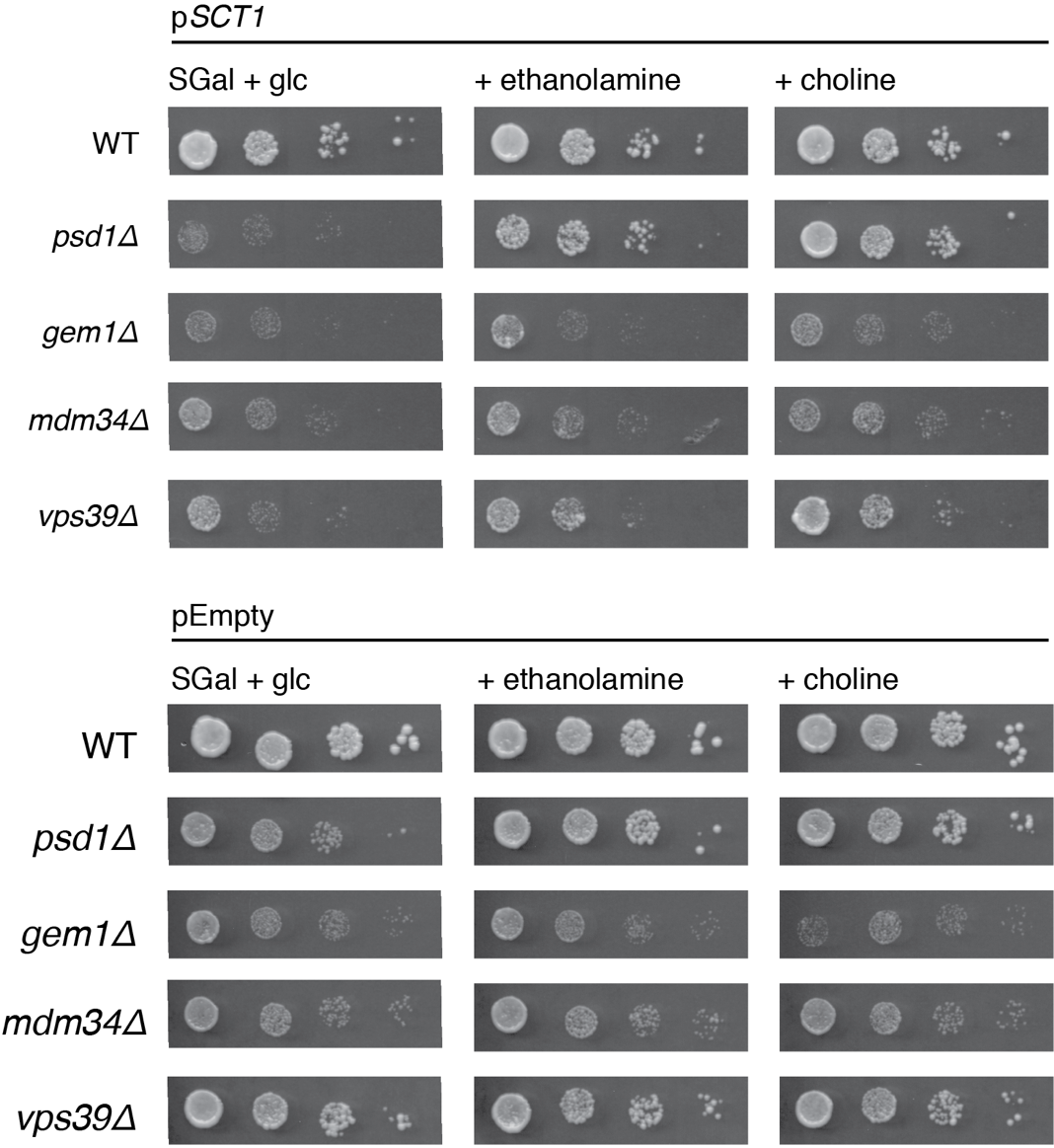


**Appendix Figure S8. Effect of ethanolamine and choline on the growth of WT and indicated mutants overexpressing *SCT1* (p*SCT1*) *vs.* control (pEmpty).**

Serial dilutions (10^-1^ – 10^-4^) of the indicated strains were spotted on SGal + 0.05% glucose and incubated for 3 days at 30°C. Ethanolamine or choline (final concentration of 1 mM) were supplied as indicated.

**Appendix Table S1. Acyl chain composition of WT and indicated mutants under *SCT1*-overexpression (p*SCT1*) *vs.* empty vector control (pEmpty)**

All strains were cultured on SGal + 0.05% glucose in the absence or presence of 1 mM ethanolamine (E^+^) or 1 mM choline (C^+^). Data is presented as mean (± SD) from n analyses.

|  | % C16:0 | % C16:1 | % C18:0 | % C18:1 | SFA / UFA | *n* |
| --- | --- | --- | --- | --- | --- | --- |
| SGal +Glc |  |  |  |  |  |  |
| WT pEmpty | 13.7 (±1.8) | 54.2 (±5.5) | 3.8 (±1.1) | 28.3 (±4.1) | 0.21 (±0.03) | *15* |
| WT p*SCT1* | 29.3 (±1.8) | 45.3 (±3.6) | 4.5 (±1.0) | 20.9 (±2.6) | 0.51 (±0.04) | *10* |
| *psd1∆* pEmpty | 18.1 (±2.0) | 51.0 (±5.8) | 5.9 (±1.9) | 25.1 (±3.2) | 0.32 (±0.05) | *15* |
| *psd1∆* p*SCT1* | 40.2 (±4.8) | 30.7 (±5.3) | 10.1 (±4.2) | 19.1 (±4.6) | 1.02 (±0.13) | *17* |
| *crd1∆* pEmpty | 15.4 (±0.5) | 49.0 (±7.4) | 4.8 (±1.3) | 30.9 (±5.7) | 0.25 (±0.03) | *4* |
| *crd1∆* p*SCT1* | 31.1 (±1.8) | 32.8 (±6.5) | 8.9 (±4.2) | 27.2 (±4.2) | 0.67 (±0.08) | *4* |
| *psd2∆* pEmpty | 14.1 (±0.7) | 50.4 (±5.9) | 4.5 (±1.3) | 30.8 (±3.9) | 0.23 (±0.03) | *3* |
| *psd2∆* p*SCT1* | 28.7 (±0.6) | 42.3 (±1.6) | 5.6 (±0.7) | 23.4 (±1.4) | 0.51 (±0.01) | *3* |
| *gem1∆* pEmpty | 13.4 (±1.0) | 50.5 (±6.2) | 4.8 (±1.6) | 31.3 (±5.3) | 0.22 (±0.03) | *7* |
| *gem1∆* p*SCT1* | 38.7 (±3.4) | 32.0 (±3.2) | 8.7 (±1.8) | 20.5 (±3.3) | 0.91 (±0.12) | *6* |
| *mmm1∆* pEmpty | 15.2 (±0.8) | 50.2 (±1.3) | 5.8 (±0.6) | 28.8 (±0.8) | 0.27 (±0.02) | *3* |
| *mmm1∆* p*SCT1* | 35.5 (±2.5) | 33.3 (±8.9) | 9.9 (±4.9) | 21.2 (±4.9) | 0.84 (±0.16) | *6* |
| *mdm10∆* pEmpty | 13.5 (±1.4) | 50.6 (±10.3) | 5.2 (±3.2) | 30.8 (5.8) | 0.23 (±0.07) | *3* |
| *mdm10∆* p*SCT1* | 35.0 (±6.0) | 36.5 (±9.8) | 8.1 (±3.1) | 20.5 (±3.2) | 0.79 (±0.25) | *6* |
| *mdm12∆* pEmpty | 16.3 (±2.2) | 54.8 (±6.6) | 5.2 (±1.2) | 27.4 (±3.8) | 0.22 (±0.05) | *4* |
| *mdm12∆* p*SCT1* | 34.9 (±2.1) | 32.4 (±3.0) | 9.3 (±1.0) | 23.3 (±3.0) | 0.80 (±0.10) | *4* |
| *mdm34∆* pEmpty | 14.1 (±1.8) | 49.9 (±5.3) | 5.4 (±1.8) | 30.7 (±3.4) | 0.24 (±0.05) | *7* |
| *mdm34∆* p*SCT1* | 35.7 (±2.4) | 37.6 (±3.3) | 7.3 (±1.2) | 19.4 (±1.8) | 0.76 (±0.09) | *7* |
| *vps13∆* pEmpty | 14.3 (±0.9) | 53.6 (±5.1) | 3.4 (±1.0) | 28.7 (±3.3) | 0.22 (±0.03) | *3* |
| *vps13∆* p*SCT1* | 29.5 (±1.0) | 40.7 (±1.6) | 6.0 (±0.5) | 23.8 (±0.5) | 0.55 (±0.04) | *3* |
| *vps39∆* pEmpty | 15.3 (±1.1) | 54.6 (±5.8) | 5.5 (±1.5) | 24.6 (±3.8) | 0.26 (±0.04) | *8* |
| *vps39∆* p*SCT1* | 33.3 (±3.5) | 39.3 (±7.1) | 7.5 (±2.9) | 19.9 (±3.8) | 0.70 (±0.12) | *8* |
| *ypt7∆* pEmpty | 13.5 (±0.9) | 48.3 (±2.6) | 5.5 (±0.4) | 32.8 (±1.4) | 0.23 (±0.02) | *3* |
| *ypt7∆* p*SCT1* | 32.5 (±3.4) | 37.7 (±3.0) | 7.2 (±1.5) | 22.6 (±3.8) | 0.66 (±0.07) | *3* |
| *3xemc∆* pEmpty | 17.9 (±1.8) | 45.0 (±4.2) | 5.3 (±0.9) | 31.8 (±2.4) | 0.30 (±0.04) | *4* |
| *3xemc∆* p*SCT1* | 23.8 (±1.0) | 43.4 (±3.6) | 5.3 (±0.9) | 27.7 (±2.4) | 0.41 (±0.04) | *4* |
| *4xemc∆* pEmpty | 23.5 (±2.9) | 43.9 (±7.1) | 6.7 (±2.5) | 25.9 (±1.8) | 0.44 (±0.11) | *5* |
| *4xemc∆* p*SCT1* | 29.2 (±4.2) | 42.1 (±3.9) | 6.0 (±1.3) | 22.7 (±1.7) | 0.55 (±0.12) | *5* |
|  |  |  |  |  |  |  |
| SGal +Glc E^+^ |  |  |  |  |  |  |
| WT pEmpty | 14.5 (±1.3) | 52.8 (±6.2) | 4.3 (±1.4) | 28.4 (±4.3) | 0.23 (±0.03) | *3* |
| WT p*SCT1* | 27.8 (±3.5) | 42.2 (±6.3) | 5.3 (±0.8) | 24.8 (±6.1) | 0.50 (±0.07) | *4* |
| *psd1∆* pEmpty | 15.1 (±1.2) | 53.8 (±2.6) | 3.3 (±0.5) | 27.8 (±3.1) | 0.23 (±0.02) | *7* |
| *psd1∆* p*SCT1* | 33.1 (±2.7) | 39.5 (±2.2) | 5.2 (±0.8) | 22.3 (±2.5) | 0.62 (±0.08) | *7* |
| *gem1∆* pEmpty | 16.2 (±3.1) | 48.8 (±3.7) | 5.3 (±1.5) | 29.7 (±5.3) | 0.28 (±0.07) | *3* |
| *gem1∆* p*SCT1* | 36.7 (±3.2) | 37.7 (±3.4) | 6.2 (±1.1) | 19.4 (±2.1) | 0.76 (±0.11) | *6* |
| *mdm34∆* pEmpty | 14.9 (±3.6) | 46.6 (±4.5) | 6.3 (±2.0) | 32.2 (±2.5) | 0.27 (±0.09) | *4* |
| *mdm34∆* p*SCT1* | 31.7 (±5.3) | 34.8 (±9.6) | 8.8 (±5.0) | 24.8 (±9.6) | 0.68 (±0.04) | *3* |
| *vps39∆* pEmpty | 14.2 (±1.0) | 56.2 (±3.8) | 5.2 (±0.5) | 24.4 (±3.6) | 0.24 (±0.02) | *5* |
| *vps39∆* p*SCT1* | 32.9 (±4.1) | 41.3 (±4.9) | 6.4 (±1.7) | 19.5 (±3.8) | 0.65 (±0.11) | *5* |
|  |  |  |  |  |  |  |
| SGal +Glc C^+^ |  |  |  |  |  |  |
| WT pEmpty | 14.1 (±1.8) | 52.6 (±5.8) | 3.1 (±1.1) | 30.3 (±4.6) | 0.21 (±0.04) | *4* |
| WT p*SCT1* | 25.6 (±1.2) | 42.1 (±3.7) | 4.0 (±0.8) | 28.3 (±3.8) | 0.41 (±0.02) | *5* |
| *psd1∆* pEmpty | 15.0 (±1.8) | 49.7 (±6.4) | 3.8 (±1.1) | 31.5 (±4.6) | 0.23 (±0.04) | *6* |
| *psd1∆* p*SCT1* | 27.5 (±3.0) | 38.8 (±4.0) | 5.1 (±2.2) | 28.7 (±4.2) | 0.48 (±0.03) | *7* |
| *gem1∆* pEmpty | 13.9 (±1.0) | 46.7 (±3.4) | 4.2 (±0.5) | 35.2 (±3.8) | 0.22 (±0.01) | *3* |
| *gem1∆* p*SCT1* | 31.6 (±1.8) | 37.5 (±2.0) | 5.6 (±0.6) | 25.4 (±1.3) | 0.59 (±0.05) | *4* |
| *mdm34∆* pEmpty | 12.3 (±1.1) | 43.6 (±5.5) | 5.5 (±1.5) | 38.5 (±5.2) | 0.22 (±0.01) | *4* |
| *mdm34∆* p*SCT1* | 30.3 (±3.0) | 35.7 (±1.2) | 6.5 (±0.3) | 27.5 (±3.8) | 0.58 (±0.07) | *3* |
| *vps39∆* pEmpty | 15.2 (±2.1) | 50.1 (±6.8) | 6.3 (±1.9) | 28.5 (±4.6) | 0.28 (±0.06) | *5* |
| *vps39∆* p*SCT1* | 24.2 (±1.5) | 40.8 (±11.1) | 6.9 (±2.9) | 28.1 (±7.2) | 0.46 (±0.08) | *5* |

**Appendix Table S2.** **Yeast strains and plasmids used in this study**

| Strain | Genotype | Source/Reference |
| --- | --- | --- |
| Wild type W303 (JNY30) | MAT**a** *leu2-3,112 trp1-1 can1-100 ura3-1 ade2-1 his3-11,15* | [1] |
| W303 *psd1Δ* (JNY1965) | W303 *psd1::NatMX6* | [1] |
| W303 *psd2Δdpl1Δ* (MA97) | W303 *dpl1::hphNT1 psd2::KanMX6* | [2] |
| W303 *psd1Δpsd2Δdpl1Δ* +Psd1(WT) (JNY1979) | W303 *psd1*::HIS3 *psd2::KanMX6 dpl1::hphNT1 ura3-1::*Psd1 | [1] |
| W303 *psd1Δpsd2Δdpl1Δ* +Psd1(Mt) (JNY1982) | W303 *psd1*::HIS3 *psd2::KanMX6 dpl1::hphNT1 ura3-1::*Psd1_MT_ | [1] |
| W303 *psd1Δpsd2Δdpl1Δ* +Psd1(ER) (JNY1984) | W303 *psd1*::HIS3 *psd2::KanMX6 dpl1::hphNT1 ura3-1::*Psd1_ER_ | [1] |
| Wild type BY4741 | MAT**a** *his3Δ1 leu2Δ0 met15Δ0 ura3Δ0* | EuroSCARF |
| *psd1Δ* | BY4741 *psd1::KanMX* | EuroSCARF |
| *psd2Δ* | BY4741 *psd2::KanMX* | EuroSCARF |
| *crd1Δ* | BY4741 *crd1::KanMX* | EuroSCARF |
| *gem1Δ* | BY4741 *gem1::KanMX* | EuroSCARF |
| *mmm1Δ* | BY4741 *mmm1::KanMX* | EuroSCARF |
| *mdm10Δ* | BY4741 *mdm10::KanMX* | EuroSCARF |
| *mdm12Δ* | BY4741 *mdm12::KanMX* | EuroSCARF |
| *mdm34Δ* | BY4741 *mdm34::KanMX* | EuroSCARF |
| *vps39Δ* | BY4741 *vps39::KanMX* | EuroSCARF |
| *vps13Δ* | BY4741 *vps13::KanMX* | EuroSCARF |
| *ypt7Δ* | BY4741 *ypt7::KanMX* | EuroSCARF |
| *3xemcΔ* (YSL6) | BY4741 *emc1::HIS5 emc2::hygMX4 emc5::kanMX4* | [3] |
| *4xemcΔ* (YSL27) | BY4741 *emc1::HIS5 emc2::hygMX4 emc3::his emc6::kanMX4* | [3] |
|  |  |  |
| Plasmid |  | **Source/Reference** |
| pYES2 (pEmpty) |  | Invitrogen |
| pYES2-*SCT1*-HH (p*SCT1*) |  | [4] |
| pYES2-*PSD1*-HA |  | [5] |
| pYPGK18 |  | [6] |
| pYPGK18-*PSD1-HA* |  | This study |

**References**

1. Friedman JR, Kannan M, Toulmay A, Jan CH, Weissman JS, Prinz WA, et al. Lipid homeostasis is maintained by dual targeting of the mitochondrial PE biosynthesis enzyme to the ER. Dev Cell. 2018;44: 261–270.e6. doi:10.1016/j.devcel.2017.11.023

2. Aaltonen MJ, Friedman JR, Osman C, Salin B, di Rago J-P, Nunnari J, et al. MICOS and phospholipid transfer by Ups2-Mdm35 organize membrane lipid synthesis in mitochondria. J Cell Biol. 2016;213: 525–534. doi:10.1083/jcb.201602007

3. Lahiri S, Chao JT, Tavassoli S, Wong AKO, Choudhary V, Young BP, et al. A conserved endoplasmic reticulum membrane protein complex (EMC) facilitates phospholipid transfer from the ER to mitochondria. PLoS Biol. 2014;12: e1001969. doi:10.1371/journal.pbio.1001969

4. De Smet CH, Vittone E, Scherer M, Houweling M, Liebisch G, Brouwers JF, et al. The yeast acyltransferase Sct1p regulates fatty acid desaturation by competing with the desaturase Ole1p. Mol Biol Cell. 2012;23: 1146–1156. doi:10.1091/mbc.E11-07-0624

5. Horvath SE, Böttinger L, Vögtle F-N, Wiedemann N, Meisinger C, Becker T, et al. Processing and topology of the yeast mitochondrial phosphatidylserine decarboxylase 1. J Biol Chem. 2012;287: 36744–36755. doi:10.1074/jbc.M112.398107

6. Vaz FM, Houtkooper RH, Valianpour F, Barth PG, Wanders RJA. Only one splice variant of the human TAZ gene encodes a functional protein with a role in cardiolipin metabolism. J Biol Chem. 2003;278: 43089–43094. doi:10.1074/jbc.M305956200
